# Chanzyme TRPM7 protects against cardiovascular inflammation and fibrosis

**DOI:** 10.1093/cvr/cvz164

**Published:** 2019-06-28

**Authors:** Francisco J Rios, Zhi-Guo Zou, Adam P Harvey, Katie Y Harvey, Ryszard Nosalski, Panagiota Anyfanti, Livia L Camargo, Silvia Lacchini, Alexey G Ryazanov, Lillia Ryazanova, Sarah McGrath, Tomasz J Guzik, Carl S Goodyear, Augusto C Montezano, Rhian M Touyz

**Affiliations:** 1 Institute of Cardiovascular and Medical Sciences, BHF Glasgow Cardiovascular Research Centre, University of Glasgow, 126 University Place, Glasgow G12 8TA, UK; 2 3rd Department of Internal Medicine, Papageorgiou Hospital, Aristotle University of Thessaloniki, Thessaloniki, Greece; 3 Department of Anatomy, Institute of Biomedical Sciences, University of São Paulo, São Paulo, São Paulo, Brazil; 4 Department of Pharmacology, Rutgers Robert Wood Johnson Medical School, New Brunswick, NJ, USA; 5 Lewis Sigler Institute of Integrative Genomics, Princeton University, Princeton, NJ, USA; 6 Centre of Immunobiology, Institute of Infection, Immunity and Inflammation, College of Medical, Veterinary and Life Sciences, University of Glasgow, Glasgow, UK

**Keywords:** Magnesium channel, Cardiac hypertrophy, Vascular inflammation, Cations

## Abstract

**Aims:**

Transient Receptor Potential Melastatin 7 (TRPM7) cation channel is a chanzyme (channel + kinase) that influences cellular Mg^2+^ homeostasis and vascular signalling. However, the pathophysiological significance of TRPM7 in the cardiovascular system is unclear. The aim of this study was to investigate the role of this chanzyme in the cardiovascular system focusing on inflammation and fibrosis.

**Methods and results:**

TRPM7-deficient mice with deletion of the kinase domain (TRPM7^+/Δkinase^) were studied and molecular mechanisms investigated in TRPM7^+/Δkinase^ bone marrow-derived macrophages (BMDM) and co-culture systems with cardiac fibroblasts. TRPM7-deficient mice had significant cardiac hypertrophy, fibrosis, and inflammation. Cardiac collagen and fibronectin content, expression of pro-inflammatory mediators (SMAD3, TGFβ) and cytokines [interleukin (IL)-6, IL-10, IL-12, tumour necrosis factor-α] and phosphorylation of the pro-inflammatory signalling molecule Stat1, were increased in TRPM7^+/Δkinase^ mice. These processes were associated with infiltration of inflammatory cells (F4/80^+^CD206^+^ cardiac macrophages) and increased galectin-3 expression. Cardiac [Mg^2+^]_i_, but not [Ca^2+^]_i_, was reduced in TRPM7^+/Δkinase^ mice. Calpain, a downstream TRPM7 target, was upregulated (increased expression and activation) in TRPM7^+/Δkinase^ hearts. Vascular functional and inflammatory responses, assessed *in vivo* by intra-vital microscopy, demonstrated impaired neutrophil rolling, increased neutrophil: endothelial attachment and transmigration of leucocytes in TRPM7^+/Δkinase^ mice. TRPM7^+/Δkinase^ BMDMs had increased levels of galectin-3, IL-10, and IL-6. In co-culture systems, TRPM7^+/Δkinase^ macrophages increased expression of fibronectin, proliferating cell nuclear antigen, and TGFβ in cardiac fibroblasts from wild-type mice, effects ameliorated by MgCl_2_ treatment.

**Conclusions:**

We identify a novel anti-inflammatory and anti-fibrotic role for TRPM7 and suggest that its protective effects are mediated, in part, through Mg^2+^-sensitive processes.

## Graphical Abstract

**Graphical Abstract:**
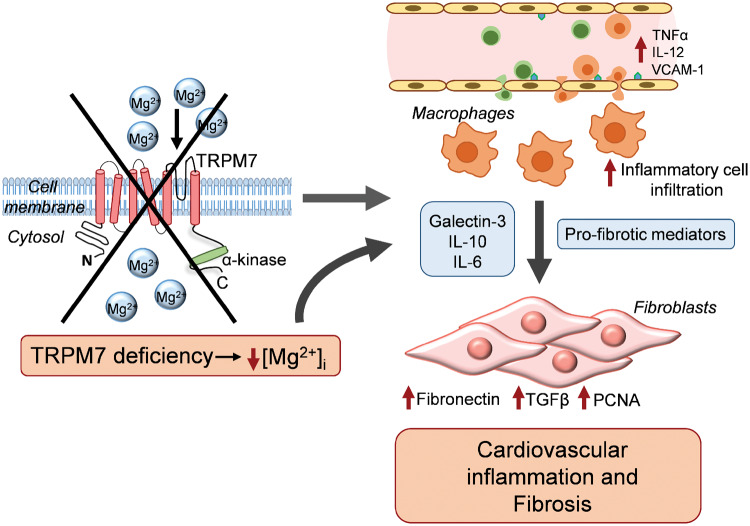

## 1. Introduction

Transient Receptor Potential Melastatin 7 cation channel (TRPM7) is a ubiquitously expressed channel fused to a C-terminal α-kinase domain located in intracellular vesicles and in the plasma membrane,^1^^,^^2^ with important roles in vascular regulation, hypertension,^3^^,^^4^ tumour progression,^5^ and immune activation.^6^^,^^7^ The channel is permeable primarily not only to Mg^2+^ but also to Zn^2+^ and Ca^2+^, and the α-kinase influences activity of downstream target proteins including annexin-1, calpain-II, myosin IIA, eukaryotic elongation factor 2-kinase (eEF2-k), and phospholipase Cγ2 (PLCγ2).^4^^,^^8^ TRPM7 has autophosphorylation residues and cleavage of the α-kinase releases fragments that bind to transcription factors resulting in epigenetic modifications.^9^ The essential and non-redundant function of TRPM7 in development was demonstrated in mice where TRPM7 and TRPM7 α-kinase knockout resulted in embryonic lethality.^10^ We showed that cells deficient in the α-kinase domain of TRPM7 exhibit a pro-inflammatory phenotype with increased expression of ICAM-1, cyclooxygenase-2 (COX-2) and plasminogen activator inhibitor-1 (PAI-1) upon aldosterone stimulation.^11^ TRPM7-kinase deficient heterozygous mice are viable, however, they develop hypomagnesaemia, vascular dysfunction and are hyper-responsive to the blood pressure-elevating effects of angiotensin II (Ang II).^3^

Chronic low-grade inflammation plays an important pathophysiological role in cardiovascular disease as evidenced by immune cell activation, production of inflammatory mediators, tissue injury, and organ damage.^12^ These inflammatory responses are accompanied by fibrosis, leading to cardiovascular complications, including heart and kidney injury and vascular dysfunction. Recruited monocytes/macrophages and lymphocytes produce pro-inflammatory mediators, such as tumour necrosis factor-α (TNFα), interleukins (ILs) (IL-6, IL-1β, IL-12), monocyte chemoattractant protein-1 (MCP-1), and reactive oxygen species (ROS) that activate transcription factors, including Stat1 and Stat3.^13^ Production of pro-fibrotic mediators including transforming growth factor-β1 (TGF-β1), PAI-1, osteoponin, and galectin-3 (Gal-3) are also implicated in cardiovascular injury and target organ damage.^14^

TRPM7 dysregulation is associated with cell injury and inflammation in various pathologies. Inhibition of TRPM7 in rheumatoid arthritis fibroblast-like synoviocytes, caused endoplasmic reticulum stress, inflammation and apoptosis^15^ and in HEK-293 cells overexpressing TRPM7, there was cell rounding and loss of adhesion with associated increased oxidative stress.^16^ TRPM7-deficient chicken B cell (DT-40) growth was arrested, which was rescued by exogenous Mg^2+^ supplementation.^17^ TRPM7 deletion in T cell lineage showed reduction in CD4+ and CD8+ T cells.^18^ Deletion of TRPM7 channel in megakaryocytes leads to macrothrombocytopenia.^19^ In macrophages, pharmacologic inhibition of TRPM7 reduced macrophage polarization towards an M2 anti-inflammatory phenotype.^20^ In the vascular system, TRPM7 inhibition reduced calcification in VSMCs^21^ and disruption of platelet TRPM7-kinase activity protected the brain from cerebral inflammation and thrombus formation.^22^ We demonstrated that TRPM7 expression is regulated by vasoactive mediators^4^^,^^23^ and that aldosterone regulates TRPM7 channel activity. In experimental hypertension, TRPM7-kinase is protective and *in vitro* data showed that the TRPM7 α-kinase deletion is associated with enhanced vascular ROS production and stimulation of pro-inflammatory signalling pathways.^3^^,^^11^

Together the above studies suggest that TRPM7 is associated with both protective and injurious effects. However, most studies were performed in *in vitro* cell-based models. The pathophysiological relevance of TRPM7 *in vivo* is elusive, especially with respect to the cardiovascular system. To address this, we deeply phenotyped heterozygous TRPM7-deficient mice (TRPM7^+/Δkinase^) focusing on inflammatory and fibrotic processes in the heart, vasculature and kidneys. We also explored the role of macrophages in these processes and investigated putative molecular mechanisms underlying TRPM7-dependent actions using bone marrow-derived macrophages (BMDM).

## 2. Methods

Please see Supplementary material online for detailed methods.

### 2.1 Animals

Animal experiments were performed in accordance with the United Kingdom Animals Scientific Procedures Act 1986 and ARRIVE Guidelines and approved by the institutional ethics review committee. We used male, 18- to 22-week-old mice. Wild-type (WT) TRPM7^+/+^ mice (C57BL/6J and SV129 mixed background) and mice heterozygous for the deletion of the TRPM7-kinase (TRPM7^+/Δkinase^), generated by the gene-targeting vector technique were studied.^10^ After study, all animals were anaesthetized by isoflurane inhalation (3%) plus 1 L/min O_2_ and then euthanized by exsanguination. TRPM7^+/Δkinase^ mice have been characterized and compared with WT mice have significantly lower Mg^2+^ concentration in plasma, urine, and bones likely due to decreased functional channel activity, and survival is reduced.^10^

### 2.2 Echocardiography

Animals were anaesthetized by isoflurane inhalation (2.5%) plus 1 L/min O_2_. Cardiac function and structure were assessed by echocardiography using an Acuson Sequoia c512 ultrasound system to acquire non-invasive 2D guided M-mode images at a 20 mm depth at the tip of the papillary muscles.

### 2.3 Plasma and urine biochemistry

Blood was collected under isoflurane anaesthesia by cardiac puncture immediately prior to sacrifice. Spot urine was collected from the bladder. Calcium, phosphate, sodium, potassium, chloride, magnesium, albumin, creatinine, plasma total cholesterol, HDL, and glucose were determined by automated methods.

### 2.4 Intra-vital microscopy to assess vascular inflammatory responses *in vivo*

Mice were injected with 20 ng of recombinant murine TNFα to induce a mild inflammatory response. Mice were anaesthetized by isoflurane inhalation (3%) plus 1 L/min O_2_. Cremaster muscle was prepared for intra-vital microscopy as described.^24^ Vessels between 25 and 35 µm were randomly selected from different areas of the tissue. Videos were recorded for 60 s. Leucocyte rolling velocity, adhesion, and transmigration were analysed.

### 2.5 Flow cytometry

Cells from heart, kidney, and spleen were labelled with cell surface markers using the following specific antibodies**:** anti-CD45-FITC (30-F11), anti-CD3-PE-Cy7 (145-2C11), anti-CD4-APC (GK1.5), anti-CD8-APC-Cy7 (53-6.7), anti-CD19-PerCP-Cy5.5 (6D5), anti-F4/80-Alexa-647 (BM8), anti-CD11c-PE-Cy7 (N418), anti-CD206-FITC (C068C2), anti-CD206-PE (C068C2), anti-CD11b-Alexa 647 (M1/70), anti-Ly-6C-APC/Cy7 (HK1.4), and anti-CD45-PE (30-F11).

### 2.6 Mg^2+^ and Ca^2+^ concentration

Total Mg^2+^ concentration in hearts and kidneys was assessed using the Magnesium Gen.2 kit. Measurement of intracellular free Mg^2+^ concentration ([Mg^2+^]_i_) and intracellular free Ca^2+^ concentration ([Ca^2+^]_i_) were assessed by magnesium green AM and Cal-520 AM, respectively.

### 2.7 Culture of BMDM and isolation of resident peritoneal macrophages

Animals were anaesthetized by isoflurane inhalation (5%) plus 1 L/min O_2_ and then euthanized by cervical dislocation. BMDM were obtained as previously described.^25^ Resident peritoneal macrophages were harvested from the peritoneal cavity of mice by lavage with cold phosphate buffered saline.

### 2.8 Culture of cardiac fibroblasts

Hearts were cut into small pieces, digested with collagenase II, and the cell suspension centrifuged. The resulting pellet was re-suspended in Dulbecco’s modified Eagle’s medium/20% fetal bovine serum, adherent cells cultured and low passage cells studied.

### 2.9 Immunoblotting

Expression of proteins was detected using specific antibodies targeted to: fibronectin, α-smooth muscle actin (α-SMA), β-actin, TGFβ1, annexin-1, calpain-II, galectin-3, proliferating cell nuclear antigen (PCNA), spectrin αII, α-tubulin, vimentin, GAPDH, Na/K ATPase, phospho-Stat3, phospho-Stat1, phospho-Smad3, and phospho-p66Shc. Protein expression was visualized using secondary fluorescence-coupled antibodies.

### 2.10 Cytosol and membrane fractionation

Translocation of annexin-1 and calpain-II from the cytosol to the membrane was assessed in cardiac tissues as a marker of TRPM7 kinase activity, since these proteins are downstream targets of TRPM7 kinase. Cytosolic and membrane fractions were obtained by ultracentrifugation.

### 2.11 Real-time reverse-transcription polymerase chain reaction

Total RNA was isolated, cDNA was generated from total RNA and real-time polymerase chain reaction performed with specific murine primers to GAPDH, fibronectin, collagen-1, TGFβ1, TNFα, IL-1β, IL-12, IL-10, Arg1, iNOS, IFN-γ, VCAM-1, MMP2, and TIMP-1.

### 2.12 Histology

Hearts, aortas, and kidneys were fixed and processed for histological examination by light microscopy (haematoxylin and eosin, picrosirius red). Fibrosis was further analysed in picrosirius red-stained samples using bright field and polarized light microscopy. Tissue fibrosis was also quantified using second harmonic generation (SHG)/two photon excitation fluorescence.

### 2.13 Immunohistochemistry

Mouse heart sections were deparaffinized and processed for antigen retrieval. Slides were incubated with the primary antibody anti-galectin-3 and secondary antibodies and counterstained with haematoxylin.

### 2.14 Enzyme-linked immunosorbent assay kits

Plasma galectin-3 levels were analysed by enzyme-linked immunosorbent assay (ELISA). Cytokine production was analysed in macrophage supernatant by ELISA (IL-6, IL-10, IL-12, TNF-α).

### 2.15 MgL and MgH vascular smooth muscle cells^26^

In some experiments, VSMCs from mice with genetically low body Mg^2+^ (MgL) or high body Mg^2+^ (MgH) were used. These mice have been well characterized.^26^^,^^27^

### 2.16 Statistical analysis

Data are presented as mean ± SEM. Two-tailed unpaired Student’s *t*-test was used when differences between two groups were analysed. Analysis of variance (ANOVA) and the Student–Newman–Keuls post-test were used to evaluate statistical significance of differences between three or more groups. Significance was assumed if *P* < 0.05.

## 3. Results

### 3.1 Plasma and urine biochemistry

Plasma electrolytes were not significantly different between groups (Supplementary material online, *Table S2*). Urinalysis showed lower levels of magnesium, potassium, phosphate, and chloride in the experimental group (Supplementary material online, *Table S2*). TRPM7^+/Δkinase^ mice had increased plasma levels of total cholesterol and reduced levels of HDL cholesterol (Supplementary material online, *Table S2*).

Galectin (Gal)-3 is a β-galactoside-binding lectin secreted by activated macrophages and is a biomarker for both cardiac inflammation and fibrosis.^28^ TRPM7^+/Δkinase^ (M7+/Δ) mice had significantly elevated levels of plasma Gal-3 (Supplementary material online, *Table S2*).

### 3.2 Tissue and cellular magnesium status in TRPM7^+/^^Δ^^kinase^ mice

**Figure 1:**
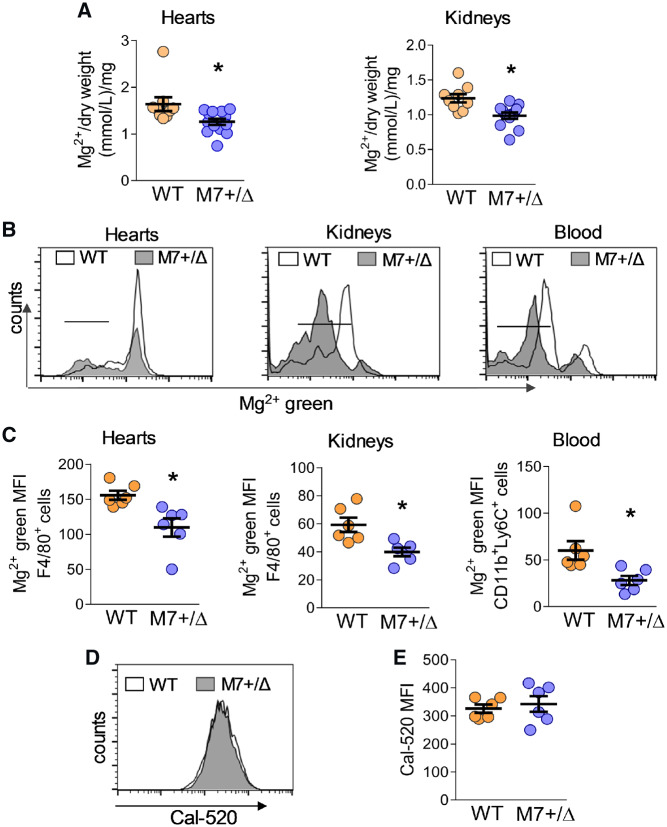
Tissue and cellular Mg^2+^ and Ca^2+^ levels in TRPM7^+/Δkinase^ mice. (*A*) Total Mg^2+^ concentration in heart and kidneys from WT (*n* = 9) and TRPM7^+/Δkinase^ (M7+/Δ) (*n* = 13) mice was assessed by colorimetric assay and normalized by dry weight. Total mononuclear leucocytes were isolated from hearts, kidneys, and blood, and labelled with magnesium green AM (Mg^2+^ green), followed by specific antibodies: anti-CD45 + anti-F4/80 for macrophage identification in heart and kidney, and anti-CD11b + anti-Ly6C for blood monocytes. (*B*) Representative flow cytometry histograms and corresponding scatter-plot graphs (*C*) showing free Mg^2+^ levels in macrophages isolated from hearts (*n* = 6/group) and kidneys and blood monocytes (*n* = 6/group). (*D, E*) Intracellular free Ca^2+^ levels in macrophages were investigated using the probe Ca-520 and analysed by flow cytometry (*n* = 6/group). Data are presented as representative flow cytometry histograms (*D*) and corresponding scatter-plot graphs (*E*). Results are mean ± SEM of mean of fluorescence intensity (MFI) of fluorescence-labelled cells assessed by flow cytometry. Statistical significance was determined by a two-tailed unpaired Student’s *t*-test. *P < 0.05 TRPM7^+/Δkinase^ (M7+/Δ, blue) vs. WT (yellow).

Total Mg^2+^ levels were lower in heart and kidneys from TRPM7^+/Δkinase^ mice vs. WT controls (*Figure 1A*). Cardiac and renal macrophages and circulating monocytes from TRPM7^+/Δkinase^ mice had 30–50% reduction in [Mg^2+^]_i_ compared with cells from WT controls (*Figure 1B, C*).

Intracellular concentration of Mg^2+^ was not associated with macrophage phenotype because there were no significant differences in [Mg^2+^]_i_ between M1 (F4/80+CD11c+) and M2 (F4/80+CD206+) macrophages in both WT and TRPM7^+/Δkinase^ mice (Supplementary material online, *Figure S2A, B*). Macrophage [Ca^2+^]i was not significantly different between groups (*Figure 1D, E*).

### 3.3 Expression of TRPM6 and TRPM7

There were no differences in gene expression for TRPM7 in hearts and kidneys between WT and TRPM7^+/Δkinase^ mice (Supplementary material online, *Figure S2C, D*). mRNA expression of TRPM6 was increased in kidneys and reduced in hearts in TRPM7^+/Δkinase^ mice (Supplementary material online, *Figure S2E, F*).

Protein expression of the kinase domain was reduced in TRPM7^+/Δkinase^ mice in hearts and kidneys (Supplementary material online, *Figure S3A, B*). This was associated with decreased TRPM7 phosphorylation and reduced expression of TRPM7 channel (Supplementary material online, *Figure S3C, D*).

### 3.4 Cardiac morphology and function

**Figure 2:**
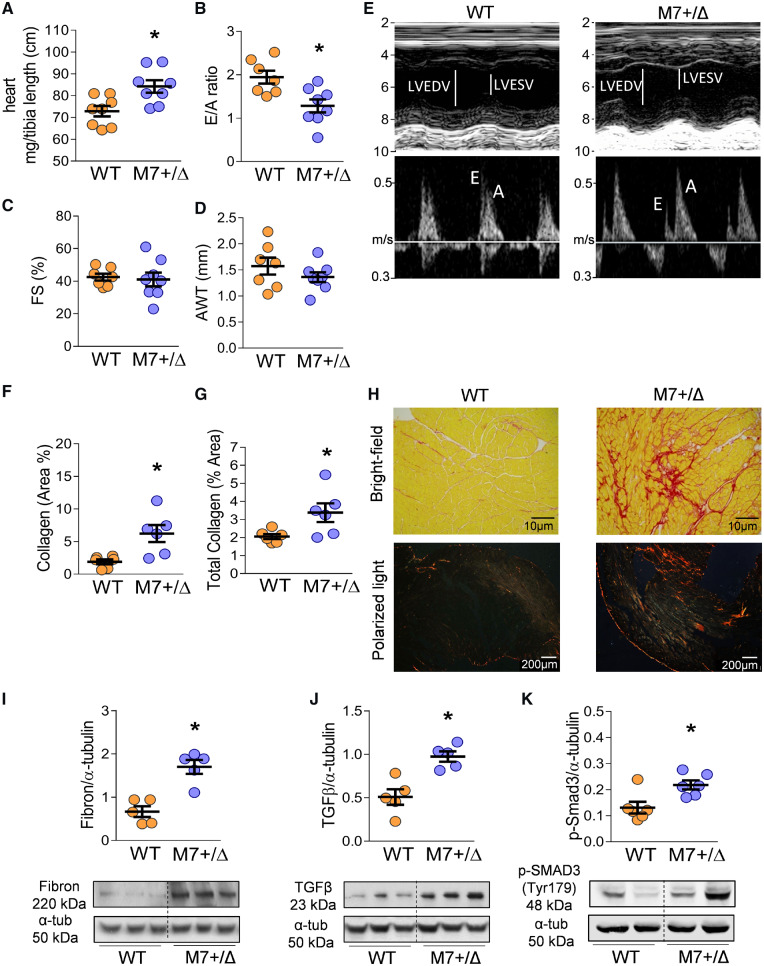
Cardiac dysfunction and fibrosis in TRPM7^+/Δkinase^ mice. (*A*) Heart weight, normalized to tibia length, in WT and TRPM7^+/Δkinase^ (M7+/Δ) mice (*n* = 8/group). (*B–E*) Echocardiography analysis showing ventricular filling velocity assessed by E/A ratio (*B*), where Early-E and late atrial-A ventricular filling velocity; (*C*) Left ventricular fractional shortening (FS%); (*D*) left ventricular AWT (WT *n* = 7, M7+/Δ *n* = 8); (*E*) Representative images by M-mode echocardiography (upper panel) and Doppler (lower panel). (*F*) Cardiac sections obtained from WT and M7+/Δ were stained with sirius red and collagen content assessed using bright field (scale bar 10 μm) and polarized light (scale bar 200 μm). Images are representative of *n* = 6/group. Collagen was analysed in bright field (*F*, *H*-upper panel) and polarized light (*G*, *H*-lower panel) and data expressed as mean ± SEM of % affected area (*n* = 6/group). Cardiac pro-fibrotic markers were assessed by immunoblotting: (*I*) fibronectin (*n* = 5/group), (J) TGFβ (*n* = 5/group), and (*K*) phospho-Smad3 (Tyr179) (*n* = 6/group). Proteins of interest were normalized to α-tubulin. Statistical significance was determined by a two-tailed unpaired Student’s *t*-test. *P < 0.05 TRPM7^+/Δkinase^ (M7+/Δ, blue) vs. WT (yellow).

TRPM7^+/Δkinase^ mice had significantly larger hearts than WT controls (*Figure 2A*). Cardiac analysis by echocardiography demonstrated reduced early (E)/late (A) [(E/A)] diastolic filling velocities, indicative of cardiac stiffening (*Figure 2B, E*). We did not observe differences in fractional shortening or anterior wall thickness (AWT) between groups (*Figure 2C–E*).

### 3.5 TRPM7 deficiency is associated with cardiac fibrosis

TRPM7^+/Δkinase^ mice had cardiac hypertrophy and increased fibrosis as evidenced by approximately three-fold increase in collagen content. Sirius red staining using bright field and polarized light microscopy, showed increased deposition of total collagen, mature collagen (red fluorescence), and immature collagen (green fluorescence) (*Figure 2F–H*, Supplementary material online, *Figure S4A, B*). Cardiac fibrosis was also observed by SHG methodology, where collagen is determined by the intensity of green autofluorescence (Supplementary material online, *Figure S4C*). Expression of cardiac fibronectin was increased ∼2.8-fold in TRPM7^+/Δkinase^ mice (*Figure 2I*). Molecular mechanisms related to fibrogenesis typically involve TGF-β production, which signals through Smad3.^29^ We found that hearts from TRPM7^+/Δkinase^ mice exhibited high expression of TGF-β (2.3-fold) and associated increased phosphorylation of Smad3 (two-fold) compared with WT mice (*Figure 2J, K*). Consistent with these findings, TRPM7 deficiency resulted in upregulation of gene expression of TGFβ1, collagen-I, and fibronectin (Supplementary material online, *Figure S4D–F*), and increased phosphorylation of p66Shc, which is involved in cardiovascular injury (Supplementary material online, *Figure S4G*).

### 3.6 Cardiac inflammation and TRPM7 signalling

**Figure 3:**
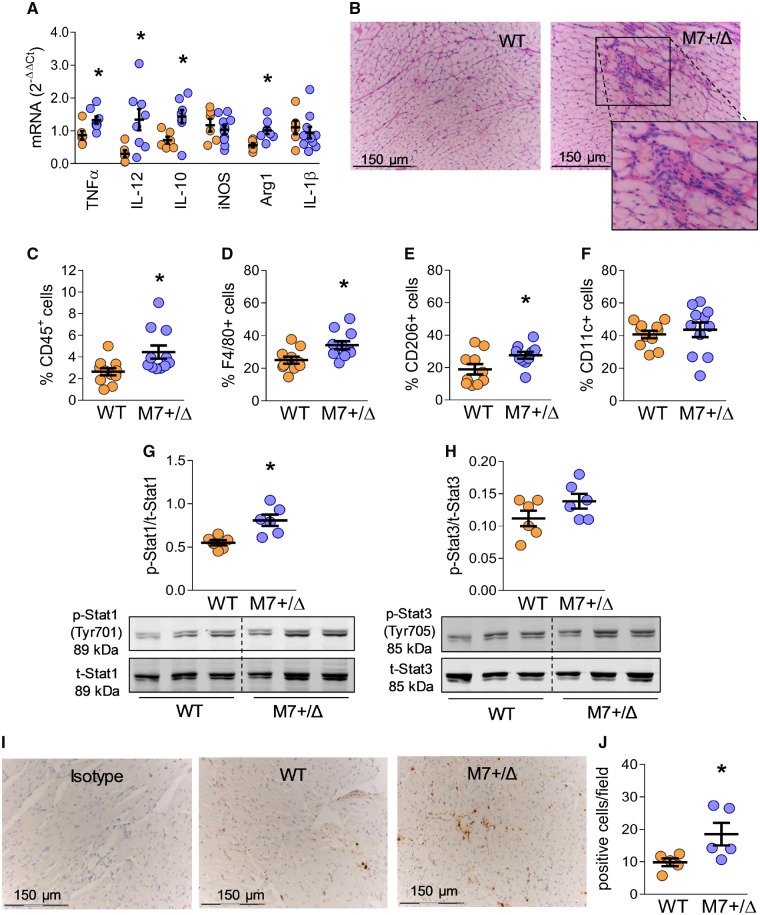
Cardiac inflammation in TRPM7^+/Δkinase^ mice. Total RNA was obtained from total cardiac tissues and gene expression for TNFα, IL-12, IL-10, iNOS, Arg-1, and IL-1β was determined by real-time PCR and normalized by GAPDH (*A*). Data are expressed in 2^−ΔΔCt^ values (WT *n* = 6, M7+/Δ *n* = 8). (*B*) Histological sections from hearts tissues from WT and TRPM7^+/Δkinase^ (M7+/Δ) mice stained with H&E showing an inflammatory infiltrate. The highlighted area in (*B*) shows the inflammatory infiltrate. Images are representative photomicrographs (*n* = 8/group), scale bar = 150 μm. Total leucocytes were isolated from hearts by enzymatic digestion and stained for flow cytometry analysis. (C) CD45+ population (total haematopoietic cells), (*D*) CD45+F4/80+ cells (macrophages); (*E*) CD45+F4/80+CD206+ (M2 macrophages); (*F*) CD45+F4/80+CD11c (M1 macrophages) (*n* = 6/group). Total lysates obtained from cardiac tissues were investigated for the expression of (*G*) phospho-Stat1 (Tyr701) and (*H*) phospho-Stat3 (Tyr705) by western blotting and normalized to total Stat1 and Stat3, respectively (*n* = 6/group). (*I*, *J*) Increased galectin-3 expression in the hearts of TRPM7^+/Δkinase^ mice. (*I*) Representative histological sections obtained from hearts from WT and TRPM7^+/Δkinase^ mice (*n* = 5/group). Controls for immunoreactivity were assessed using the isotype only. (*J*) Corresponding scatter-plot graphs indicating galectin-3 expression by immunohistochemistry. Scale bar = 150 μm (*n* = 5/group). Results are mean ± SEM. Statistical significance was determined by a two-tailed unpaired Student’s *t*-test. *P < 0.05 TRPM7^+/Δkinase^ (M7+/Δ, blue) vs. WT (yellow).

Fibrosis is often associated with inflammation, and may be a consequence of dysregulated inflammatory responses. Hearts from TRPM7^+/Δkinase^ mice exhibited increased gene expression of TNFα and IL-12, potent pro-inflammatory cytokines and of IL-10 and Arg1, important proteins in the resolution phase of inflammation, tissue repair, and fibrosis (*Figure 3A*). TRPM7^+/Δkinase^ mice had increased cardiac inflammatory cell infiltration visualized by HE staining and confirmed by the increased presence of CD45+ cells (*Figure 3B, C*), and F4/80+CD206+ macrophages (*Figure 3D–F*), a phenotype present in scar tissue and important in resolution of inflammation and fibrosis. They also had an increase content of CD3+ cells and CD8+ T cell but not CD4+ T cells or B cells (Supplementary material online, *Figure S5A–D*). Phosphorylation of the transcription factors Stat1 and Stat3 is critically involved in inflammation and fibrosis.^13^ Cardiac phosphorylation of Stat1, but not Stat3, was increased in TRPM7^+/Δkinase^ mice compared with WT animals (*Figure 3G, H*). In addition, cardiac expression of galectin-3 was increased in TRPM7^+/Δkinase^ mice (*Figure 3I, J*).

**Figure 4:**
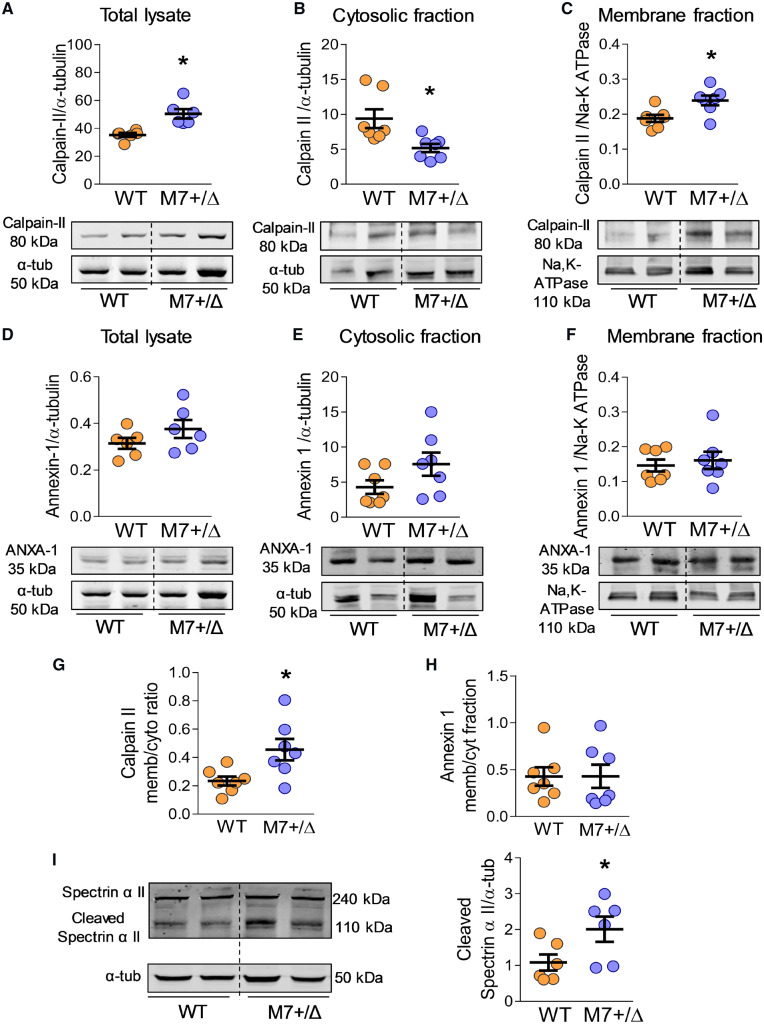
Cardiac calpain-II and annexin-1, downstream targets of TRPM7, in TRPM7^+/Δkinase^ mice. Cardiac expression of Calpain-II and Annexin-1 (ANXA-1) in total heart lysate (*A*, *D*) (*n* = 6/group), cytosolic (*B*, *E*) and membrane fractions (*C*, *F*) (*n* = 7/group) assessed by immunoblotting. Cytosolic and membrane fractions from total cardiac tissues were obtained by ultracentrifugation. Protein expression in total lysate and cytosolic fractions were normalized to α-tubulin and the membrane fraction was normalized to Na-K ATPase content. The ratio of membrane: cytosolic calpain-II (*G*) and annexin-1 (*H*) is used as an index of cytosol to membrane translocation. (*I*) Total lysates from cardiac tissues were used to assess expression of spectrin α II, total (240 kDa) and cleaved forms (110 kDa), by western blotting and further normalized to α-tubulin (*n* = 6/group). Results are presented as representative immunoblots and corresponding scatter-plot graphs. Data are means ± SEM. Statistical significance was determined by a two-tailed unpaired Student’s *t*-test *P < 0.05 TRPM7^+/Δkinase^ (M7+/Δ, blue) vs. WT (yellow).

Annexin-1 and calpain-II (m-calpain), which translocate to the cell membrane upon activation, are known targets for TRPM7 α-kinase.^16^^,^^30–33^. Cardiac expression of calpain-II and translocation from the cytosol to the membrane were increased in TRPM7^+/Δkinase^ mice as evidenced by increased membrane: cytosolic expression (*Figure 4A–C, G*). Cardiac expression and activity of annexin-1 were unaltered in hearts from TRPM7^+/Δkinase^ mice (*Figure 4D–F, H*).

Calpain-II activity was further evaluated by assessing cleavage of spectrin α II (α-fodrin), a cytoskeletal protein sensitive to calpain-II proteolytic activity. As shown in *Figure 4I*, spectrin αII cleavage was increased in hearts from TRPM7^+/Δkinase^ mice.

### 3.7 Increased leucocyte transmigration and inflammation in vessels in TRPM7^+/^^Δ^^kinase^ mice

**Figure 5:**
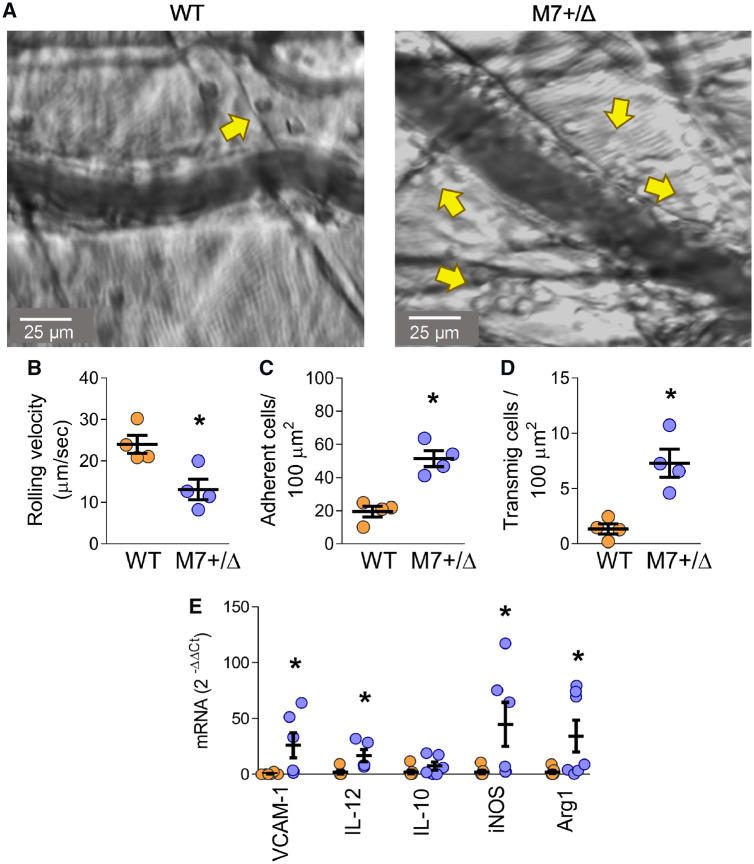
Increased vascular inflammation in TRPM7^+/Δkinase^ mice. (*A*) Intra-vital images obtained from cremasteric microvessels 3 h after intrascrotal injection of TNFα (20 ng/mL). Images are representative bright field of WT (left panel) and TRPM7^+/Δkinase^ (M7+/Δ) (right panel) animals. Yellow arrows indicate transmigrated cells, scale bars = 25 μm. Images were recorded for 60 s from at least eight vessels from each animal (*n* = 4/group) and analysed for (*B*) rolling velocity, (*C*) number of adherent cells, and (*D*) number of transmigrated cells and normalized by 100 μm^2^. Data are presented as mean ± SEM. (*E*) Total RNA was obtained from aorta and gene expression for VCAM-1, IL-12, IL-10, iNOS, and Arg-1 was determined by real-time PCR and normalized to GAPDH expression. Data are expressed in 2^−ΔΔCt^ values (*n* = 6–7/group). Data are means ± SEM. Statistical significance was determined by a two-tailed unpaired Student’s *t*-test. *P < 0.05 TRPM7^+/Δkinase^ (M7+/Δ) (blue) vs. WT (yellow).

To assess vascular functional and inflammatory responses in TRPM7^+/Δkinase^ mice, we performed intra-vital microscopy in cremasteric venules (*Figure 5A*). TRPM7^+/Δkinase^ mice showed reduced rolling velocity (by 55%) (*Figure 5B*), with strong adhesion (200%) (*Figure 5C*), and neutrophil transmigration into the vascular wall (450%) (*Figure 5D*) compared with WT mice. Together, these findings demonstrate that TRPM7 deficiency leads to a pro-inflammatory condition in vessels characterized by neutrophil: endothelial adhesion and transmigration into the vascular media and perivascular tissue.

The inflammatory phenotype was also observed in aorta from TRPM7^+/Δkinase^ mice, which had higher mRNA expression of VCAM-1 (25-fold), IL-12 (6.8-fold), iNOS (12-fold), and Arg1 (18-fold) (*Figure 5E*). Markers of vascular fibrosis including collagen deposition (Supplementary material online, *Figure S6A–C*) and gene expression for collagen-1, fibronectin, TGFβ1, and TIMP1/MMP2 (Supplementary material online, *Figure S6D–G*) were not different between TRPM7^+/Δkinase^ and WT mice.

### 3.8 TRPM7 deficiency is associated with inflammatory/immune cell infiltration in kidneys

**Figure 6:**
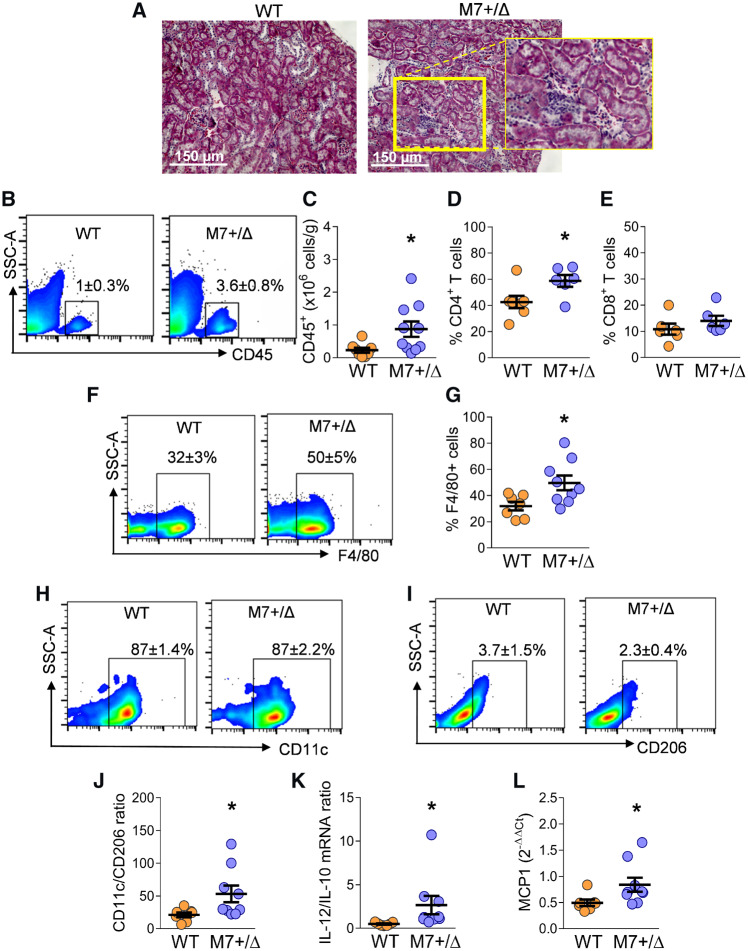
Renal inflammation in TRPM7^+/Δkinase^ mice. (*A*) Representative photomicrographs of histological kidney sections stained with H&E (*n* = 8/group). The highlighted area in (*A*) shows the inflammatory infiltrate. Scale bar = 150 μm. Total leucocytes were isolated from kidneys by enzymatic digestion and stained for flow cytometry analysis. Results are shown as representative plots and as mean ± SEM (WT *n* = 8, M7+/Δ *n* = 10) of (*B*, *C*) CD45+ population (total haematopoietic cells); (*D*) CD45+CD3+CD4+ cells (CD4 T lymphocytes); (*E*) CD45+CD3+CD8+ cells (CD8 T lymphocytes) (*n* = 6/group); (*F*, *G*) CD45+F4/80+ cells (macrophages); (*H*) CD45+F4/80+CD11c+ (M1 macrophages); (*I*) CD45+F4/80+CD206+ (M2 macrophages); (*J*, *K*) CD11c to CD206 ratio (WT *n* = 7, M7+/Δ *n* = 9) and IL-12 to IL-10 mRNA ratio; (*L*) MCP-1 mRNA gene expression (WT *n* = 7, M7+/Δ *n* = 9). Statistical significance was determined by a two-tailed unpaired Student’s *t*-test. *P < 0.05 TRPM7^+/Δkinase^ (M7+/Δ, blue) vs. WT (yellow). SSC-A, Side-Scatter.

Uncontrolled inflammatory cell migration into tissues is an important process underlying renal damage. Kidneys from TRPM7^+/Δkinase^ mice exhibited increased inflammatory infiltration as determined by haematoxylin-eosin staining (*Figure 6A*), which was confirmed by flow cytometry, that showed a approximately four times increase in the total number of haematopoietic CD45+ cells in kidneys from TRPM7^+/Δkinase^ mice vs. WT controls (*Figure 6B, C*). Characterization of the immune cell infiltrate showed that kidneys from TRPM7^+/Δkinase^ mice had increased CD4+ T lymphocytes and macrophages (F4/80+ cells) (*Figure 6D–G*). The B cell population was reduced in kidneys from TRPM7^+/Δkinase^ animals (Supplementary material online, *Figure S7A, B*). Renal macrophages from TRPM7^+/Δkinase^ were typically pro-inflammatory evidenced by the increased ratio of CD11c/CD206 expression markers (*Figure 6H–J*) and IL-12/IL10 mRNA (*Figure 6K*) and higher expression of MCP-1 mRNA (*Figure 6L*), which is a chemokine involved in monocyte migration. mRNA expression for TNFα, IFNγ, IL-1β, and fibrotic markers collagen-I, TGFβ, and fibronectin was similar in kidneys from WT and TRPM7^+/Δkinase^ animals (Supplementary material online, *Figure S7C–J*). Together these data indicate that kidneys from TRPM7^+/Δkinase^ animals have low-grade inflammation indicated by the high inflammatory infiltration, M1 macrophages and increased population of CD4+ T cells. However, kidney injury at this stage does not seem to be associated with significant fibrosis.

### 3.9 Systemic inflammation in TRPM7^+/^^Δ^^kinase^ mice

Inflammatory infiltration in organs is usually associated with systemic inflammation. We found a 45% reduction in spleen weight in TRPM7^+/Δkinase^ animals (Supplementary material online, *Figure S8A, B*) which is typically observed in auto-immune disorders.^34^ Spleens from TRPM7^+/Δkinase^ animals had a higher total number of immune cells (3.7 × 10^8^ cells/mg) compared with WT (2.0 × 10^8^ cells/mg) (Supplementary material online, *Figure S8C*) and higher percentage of CD4+ and CD8+ T lymphocytes (Supplementary material online, *Figure S8D, E*). TRPM7^+/Δkinase^ exhibited higher frequency of splenic macrophages and similar to what was observed in kidneys macrophages showed a pro-inflammatory M1 phenotype, with an increase in the ratio of CD11c/CD206 expression markers (Supplementary material online, *Figure S8F, G*). B cell population was similar in both groups (Supplementary material online, *Figure S8H*). Spleens from TRPM7^+/Δkinase^ animals also exhibited increased mRNA expression of the pro-inflammatory markers IFN-γ and IL-6 and reduced expression of the anti-inflammatory cytokine IL-10 (Supplementary material online, *Figure S8I–K*). Protein expression of pro-fibrotic markers fibronectin and TGFβ was also increased in splenic tissues from TRPM7^+/Δkinase^ animals (Supplementary material online, *Figure S8L, M*). Blood monocytes can be identified as pro- or anti-inflammatory by high or low expression of marker Ly6C, respectively.^35^ We found that TRPM7^+/Δkinase^ and WT animals presented similar frequencies of Ly6C^high^ and Ly6C^low^ cells (Supplementary material online, *Figure S9A–C*).

### 3.10 TRPM7-deficient BMDM induce a fibrotic and proliferative phenotype in cardiac fibroblasts: effects of MgCl_2_ treatment

**Figure 7:**
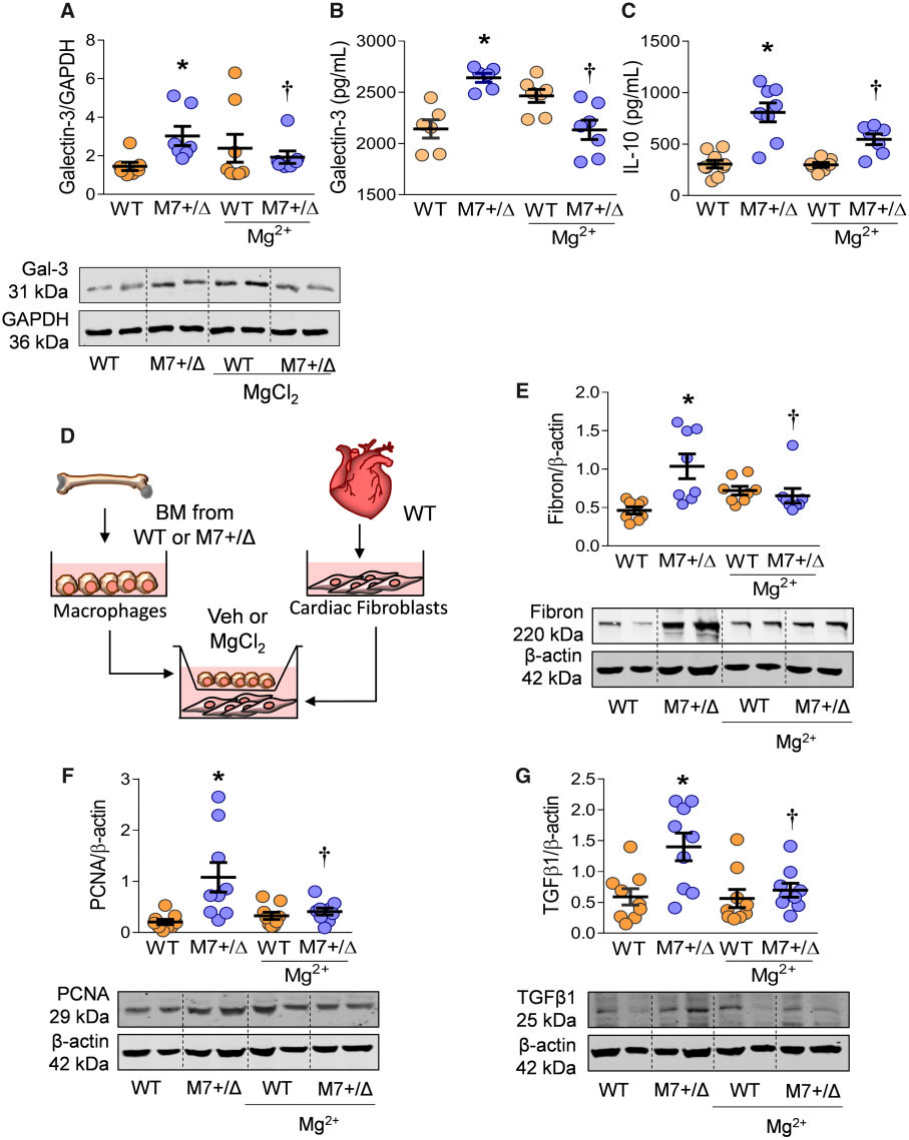
TRPM7^+/Δkinase^ macrophages induce a fibrotic phenotype in cardiac fibroblasts from WT mice: effects of MgCl_2_ treatment. BMDM were differentiated from TRPM7^+/Δkinase^ (M7+/Δ) and WT mice and treated with MgCl_2_ (10 mM) for 24 h. (*A*) Total cell lysate was analysed for Galectin-3 expression by immunoblotting and normalized to GAPDH protein expression. The production of (*B*) Galectin-3, and (*C*) IL-10 was analysed in the macrophage supernatant by ELISA (*n* = 7–8/group). (*E*) Primary culture cardiac fibroblasts from WT mice were co-cultured in transwell system with macrophage from M7+/Δ and WT animals, treated or not with MgCl_2_. After 48 h stimulation, the total cell lysate was obtained from cardiac fibroblasts and expression of (*F*) fibronectin (*n* = 8/group) (*G*) PCNA (*n* = 9/group), and (*H*) TGFβ (*n* = 9/group) was analysed by western blotting and normalized to β-actin. Data are presented as representative immunoblots and corresponding scatter-plot graphs. Results are mean ± SEM of M7+/Δ (blue) and WT (yellow). Statistical significance was determined by one-way ANOVA using the Student–Newman–Keuls post-test. *P < 0.05 for TRPM7^+/Δkinase^ (M7+/Δ) compared with WT mice. ^**†**^TRPM7^+/Δkinase^ treated with MgCl_2_ vs. untreated TRPM7^+/Δkinase^.

BMDM from TRPM7^+/Δkinase^ mice showed increased production of Gal-3, IL-10, and IL-6 as determined by western blot from the cell lysate and ELISA from the cell supernatant (*Figure 7A–C*, Supplementary material online, *Figure S10D*). These cells also had increased expression of calpain-II (Supplementary material online, *Figure S10A, B*). Treatment of macrophages with MgCl_2_ increased [Mg^2+^]_i_ (Supplementary material online, *Figure S10C, D*) and reduced the production of Gal-3 and IL-10 only in macrophages from TRPM7^+/Δkinase^ mice (*Figure 7A–C*). Similar to BMDM, isolated resident peritoneal macrophages from TRPM7^+/Δkinase^ animals produced higher concentrations of IL-10, IL-6, and TNFα compared with WT animals (Supplementary material online, *Figure S11A–D*).

To evaluate the functional significance of TRPM7-deficient macrophages, we assessed pro-fibrotic effects of TRPM7^+/Δkinase^ macrophages on WT cardiac fibroblasts in a co-culture system using the transwell approach (*Figure 7E*). As shown in *Figure 7F–H* (Supplementary material online, *Figure S12A, B*) co-culture of cardiac fibroblasts with TRPM7^+/Δkinase^ macrophages increased expression of fibronectin, PCNA, and TGFβ, proteins involved in the extracellular matrix and cell proliferation. Importantly, these effects were ameliorated when macrophages were treated with MgCl_2_. Similar effects were observed when cardiac fibroblasts were treated only with supernatants of macrophages (Supplementary material online, *Figure S13A–E*), indicating that macrophages from TRPM7^+/Δkinase^ mice produce soluble factors that promote a pro-fibrotic phenotype in cardiac fibroblasts through Mg^2+^-dependent mechanisms.

### 3.11 Increased expression of pro-inflammatory and pro-fibrotic markers in vascular smooth muscle cells from mice with low Mg^2+^ (MgL)

To further confirm the relationship between low Mg^2+^ and vascular fibrosis and inflammation, we studied another experimental model, specifically VSMCs from mice with genetically low Mg^2+^ (MgL) or high Mg^2+^ (MgH). These mice have been well characterized.^26^^,^^27^ As shown in Supplementary material online, *Figure S14*, expression of fibronectin and TGFβ was significantly greater in MgL vs. MgH cells.

## 4. Discussion

Major findings from our study show that TRPM7^+/Δkinase^ mice exhibit cardiovascular inflammation and fibrosis, processes associated with abnormal macrophage activation and intracellular Mg^2+^ deficiency. The pro-fibrotic and pro-inflammatory effects of reduced TRPM7 function are mediated in part through Mg^2+^-dependent mechanisms, because cellular Mg^2+^ treatment ameliorated deleterious effects in cardiac fibroblasts exposed to TRPM7^+/Δkinase^ BMDM. Together our data suggest that TRPM7 protects against inflammatory responses and fibrosis in the cardiovascular system.

TRPM7 and its homologue TRPM6 are unusual bifunctional molecules. The channel is permeable primarily to Mg^2+^ as well as Ca^2+^ and Zn^2+^, whereas the α-kinase domain activates downstream target proteins involved in cytoskeleton organization, cell proliferation, inflammatory responses, and vascular contraction among other properties.^1^^,^^2^ While some studies suggested that a functional α-kinase domain is necessary for TRPM7 channel-mediated cation influx,^10^^,^^36^^,^^37^ others reported that the α-kinase domain is not required.^6^^,^^11^ Here, we show that TRPM7^+/Δkinase^ cells have lower intracellular Mg^2+^ levels compared with WT control counterparts, supporting the notion that the kinase domain may influence the channel domain. This was further supported by our findings that phosphorylation of TRPM7 channel was reduced in TRPM7^+/Δkinase^ mice. These findings are in line with electrophysiology studies that showed decreased TRPM7 current in mast cells from TRPM7^+/Δkinase^ mice.^10^

TRPM7-deficient mice exhibited an inflammatory phenotype characterized by increased production of chemokines and pro-inflammatory cytokines and increased migration of macrophages into the heart, vessels, and kidneys. In addition to systemic inflammation, TRPM7^+/Δkinase^ mice had hypercholesterolaemia with reduced HDL levels. The exact cause of this metabolic derangement is unclear, but may relate to impaired cellular Mg^2+^ homeostasis and altered lipid metabolism associated with liver inflammation. Although we did not examine the liver in our study, previous studies showed an association between Mg^2+^, NF-κB/NLRP3 inflammasome activation and lipid metabolism.^38^

Monocytes/macrophages are cells of the innate immune system. Uncontrolled activation promotes tissue injury and fibrosis. These cells differentiate into classically activated M1 macrophages that are associated with pro-inflammatory responses and tissue destruction, whereas alternative activated or M2 macrophages regulate the inflammatory response and tissue repair, depending on the microenvironment.^39^ Both macrophage populations co-exist in tissues and participate in fibrogenesis.^39^ Our results show a significant inflammatory response in the heart and kidneys of TRPM7^+/Δkinase^ mice, characterized primarily by increased populations of macrophages, CD4+ T cells and CD8+ T cells. Whereas macrophages in the heart were mainly of the CD206-expressing M2 phenotype, those in the kidney were primarily CD11c-expressing M1 macrophages. M1 macrophages release mediators, including ILs, TNFα, and reactive oxygen species, which stimulate inflammatory signalling pathways causing tissue injury. In contrast, M2 macrophages release factors that modulate both inflammation and fibrosis including IL-10, galectin-3, and TGF-β.^39^

In addition to altered T cells, B cell status was modified in TRPM7-deficient mice. In particular, the number of B cells was reduced in kidneys from TRPM7^+/Δkinase^ mice. This may relate to impaired maturation of B cells because TRPM7 has been shown to play an important role in B cell maturation.^40^ Since B cells are responsible for protective antibody production, the reduced levels in TRPM7-deficient mice might further contribute to renal inflammation and injury. The effect seems to be tissue-specific because there were no differences in the number of B cells in spleens and hearts between WT and TRPM7^+/Δkinase^ mice.

Our findings identified distinct inflammatory/fibrogenic responses in the heart and kidney in TRPM7-deficient mice. While exact reasons for this are unclear, a number of factors may play a role. Firstly, the origin of macrophages may be important because those in fibrotic hearts derive from the splenic reservoir and bone marrow^41^ whereas macrophages from kidneys derive only from the bone marrow.^35^ Secondly, inflammation and fibrosis are dynamic processes and perhaps the disparity in the heart and kidney reflects different phases of tissue injury. Finally, the presence of TRPM6 in the kidney but not in the heart, may modulate TRPM7 function and inflammatory responses. In support of this, we found increased TRPM6 expression in kidneys but not in hearts from TRPM7^+/Δkinase^ mice.

The inflammatory response was not restricted to immune cells since TRPM7-deficient mice had increased vascular expression of the adhesion molecule ICAM-1, which promotes adhesion of pro-inflammatory neutrophils. The functional significance of this was assessed in vessels *in situ* in real time by intra-vital microscopy, which showed that TRPM7^+/Δkinase^ mice exhibited reduced neutrophil rolling, increased neutrophil: endothelial adhesion and enhanced transmigration into the vascular media and perivascular tissue in response to low-dose TNFα. These processes, together with activation of macrophages in the vascular wall are critically involved in triggering the vascular response in atherosclerosis, hypertension, and diabetes. The importance of TRPM7 in neutrophil migration and infiltration has previously been demonstrated in human neutrophil cell lines^42^ and sepsis-induced lung injury in rats.^43^

Chronic inflammation predisposes to fibrosis and target organ damage. In the heart, fibrosis causes cardiac enlargement and stiffening, ventricular dysfunction and ultimately cardiac failure. These processes are accelerated with aging. TRPM7-deficient mice exhibited cardiac hypertrophy and abnormal function assessed by echocardiography. These changes were associated with increased collagen deposition, enhanced expression of fibronectin and TGFβ and phosphorylation of Smad3, which is downstream of TGFβ, indicating marked cardiac fibrosis. Molecular mechanisms whereby TRPM7-kinase deficiency causes fibrosis may relate to activated macrophages as well as dysregulation of downstream TRPM7 downstream targets, especially annexin-1 and calpain-II.^4^^,^^44^ Annexin-1 is anti-inflammatory^45^ whereas calpain-II has pro-inflammatory and pro-fibrotic properties.^46^ Calpain activation is directly involved in cardiac hypertrophy and inflammation through pathways involving NF-kB, NFAT, and TGF-β.^47^ We found increased calpain-II activity in TRPM7^+/Δkinase^ mice as evidenced by increased membrane: cytosol expression and cleavage of spectrin α-II. These data are in contrast to our previous findings and those of others, which demonstrated blunted calpain activity when TRPM7 is downregulated.^11^ However, most previous studies were performed in cell-based systems, which do not reflect intact systems *in vivo*. Moreover, increased calpain-II activity may be secondary to the inflammatory state in TRPM7^+/Δkinase^ mice.

Levels of Gal-3 were increased in plasma and cardiac tissue of TRPM7-deficient mice. Gal-3 is produced mainly by macrophages and activates fibroblasts to proliferate and produce collagen-I, TGFβ, and other pro-fibrotic markers such as α-smooth muscle actin (α-SMA).^14^^,^^48^ Clinically plasma Gal-3 is a biomarker of heart failure and cardiac fibrosis^28^ and in rats injected with intra-pericardial Gal-3 the heart undergoes remodelling and fibrosis.^49^ Gal-3 is also involved in tissue inflammatory cell infiltration and leucocyte recruitment to tissues.^50^ Differentiated macrophages from TRPM7^+/Δkinase^ mice produced higher levels of Gal-3, IL-6, and IL-10, which are also involved in cardiac fibrosis. Gal-3 mediates effects through Stat1 and Stat3.^13^^,^^51^^,^^52^ In TRPM7^+/Δkinase^ hearts, phosphorylation of Stat1, but not Stat3, was significantly increased. Whether this is due to a direct effect of decreased TRPM7 kinase activity or secondary to macrophage activation remains unclear. The pro-fibrotic phenotype of TRPM7^+/Δkinase^ macrophages is further evidenced by the findings that these cells induced expression of fibronectin and PCNA in cardiac fibroblasts from WT animals in a Mg^2+^-dependent manner.^7^

Our study contributes to the growing evidence that TRPM7 is a new player in immune cell regulation and inflammation as recently highlighted by Nadolni and Zierler^53^and Santoni *et al*.^54^ To our knowledge, we provide the first comprehensive evidence defining a regulatory role for TRPM7 in cardiovascular inflammation and fibrosis. While we identify TRPM7 as being protective in line with its fundamental and non-redundant role in cellular physiology and viability,^2^^,^^10^, others suggest that TRPM7 activation causes dysregulated immune responses and inflammation and that TRPM7 inhibition may have therapeutic potential in pro-inflammatory diseases and immune hypersensitivity.^43^^,^^55^ These discrepancies likely depend on the relative contributions of TRPM7 channel vs. kinase and highlight the complexity of the system.

In summary, our data show a distinct pro-inflammatory and pro-fibrotic cardiovascular and renal phenotype in TRPM7^+/Δkinase^ mice, processes linked to macrophage activation, increased signalling through Smad3, calpain-II, and Stat1 and cellular hypomagnesaemia. Taken together, these findings suggest that TRPM7 has anti-inflammatory and anti-fibrotic functions, at least in the model studied. We define a novel and important protective role of TRPM7 in cardiovascular inflammation, organ injury and cardiac fibrosis, cellular responses that involve immune cell activation mediated, in part, through Mg^2+^-dependent processes.

## Supplementary material

Supplementary material is available at *Cardiovascular Research* online.

## Authors’ contributions

F.J.R. designed the study, performed experiments, analysed data, prepared the figures, and wrote the manuscript; Z.-G.Z., A.P.H., K.Y.H., R.N., P.A., L.L.C., S.L., and S.M. performed experiments; L.V.R. and A.G.R. provided the TRPM7+/Δ mice; C.S.G. and T.J.G. critical discussion; A.C.M. performed experiments, designed the study, experimental supervision critical discussion; R.M.T. general supervision, designed the study, supported experiments, critical discussion, preparation and submission of the manuscript.

## Supplementary Material

cvz164_Supplementary_Data

## Notes

**Time for primary review: 59 days**

## Notes

Translational Perspectives Mg^2+^ is an essential cation that influences vascular tone, cardiac contractility, and immune cell function. Mg^2+^ homeostasis is regulated by cation transporters, particularly TRPM7. Clinically, hypomagnesaemia is associated with cardiovascular disorders including arrythmias, hypertension, and vascular calcification, processes that involve vascular dysfunction, inflammation and fibrosis. Molecular mechanisms driving Mg^2+^-related cardiovascular disease are elusive, but altered TRPM7 function may be important. Our study revealed that downregulation of TRPM7 and consequent intracellular Mg^2+^ deficiency promotes cardiovascular inflammation and fibrosis. These findings suggest that the TRPM7/Mg^2+^ pathway is cardiovascular protective and may be a novel therapeutic target in cardiovascular disease.
