# Supplementary material for: Chanzyme TRPM7 protects against cardiovascular inflammation and fibrosis
Source: Cardiovasc Res. 2019 Jun 28;116(3):721–35. doi: 10.1093/cvr/cvz164 (PMC7252442; doi:10.1093/cvr/cvz164)
Supplement: cvz164_Supplementary_Data [file cvz164_supplementary_data.zip › cvz164-suppl_data/SUPPLEMENTAL-CLEAN.docx]

**SUPPLEMENTAL TEXT**

**Chanzyme TRPM7 protects against cardiovascular inflammation and fibrosis**

Francisco J. Rios^1^, Zhi-guo Zou^1^, Adam P. Harvey^1^, Katie Y. Harvey^1^, Ryszard Nosalski^1^ Panagiota Anyfanti^2^, Livia L. Camargo^1^, Silvia Lacchini^3^, Alexey G Ryazanov^4^, Lillia Ryazanova^5^, Sarah McGrath^6^, Tomasz J. Guzik^1^, Carl S. Goodyear^6^, Augusto C. Montezano^1^, and Rhian M. Touyz^1^.

^1^ Institute of Cardiovascular and Medical Sciences, BHF Glasgow Cardiovascular Research Centre, University of Glasgow, Glasgow, United Kingdom.

^2^ 3rd Department of Internal Medicine, Papageorgiou Hospital, Aristotle University of Thessaloniki, Thessaloniki, Greece. ^3^ Department of Anatomy, Institute of Biomedical Sciences, University of São Paulo, São Paulo, São Paulo, Brazil. ^4^ Department of Pharmacology, Rutgers Robert Wood Johnson Medical School, USA. ^5^Lewis Sigler Institute of Integrative Genomics, Princeton University, Princeton, USA. ^6^Centre of Immunobiology, Institute of Infection, Immunity and Inflammation, College of Medical, Veterinary and Life Sciences, University of Glasgow, Glasgow, United Kingdom.

**Correspondence:**

Rhian M Touyz MBBCh, PhD, FRCP, FRSE.

Institute of Cardiovascular & Medical Sciences

BHF Glasgow Cardiovascular Research Centre

University of Glasgow

126 University Place, Glasgow G12 8TA

Tel: + 44 (0)141 330 7775/7774; Fax: + 44 (0)141 330-3360

Email: [Rhian.Touyz@glasgow.ac.uk](mailto:Rhian.Touyz@glasgow.ac.uk)

**1. Supplemental Methods**

**1.1. Animals**

We used male, 18-22 week old mice. Wild type (WT) mice (C57BL/6J and SV129 mixed background) and mice heterozygous for the deletion of the TRPM7-kinase (TRPM7^+/Δkinase^), generated by the gene-targeting vector technique^1^. The mutation was generated by inserting a Neo gene cassette between the exons 32-36 of the kinase. As a result, the TRPM7 protein is truncated immediately upstream of the alpha-kinase domain. Therefore, using the reverse transcription–PCR analysis we are able to differentiate WT (TRPM7+/+) and heterozygous (TRPM7+/Δ) animals. Animals are littermates generated in the mix background C57Bl/6 and SV129. To generate WT and TRPM7+/Δ, breeding pairs were formed with male WT and female TRPM7+/Δ. The offspring can be either homozygous TRPM7+/+ (WT) or heterozygous (TRPM7+/Δ), which are determined by genotyping after 3 weeks old. Mice were maintained in controlled room temperature (25°C) with a 12-h light/dark cycle and food and water *ad libitum.* Homozygous TRPM7Δkinase mice are embryonic lethal, whereas heterozygous mice are viable ^1^. Genotyping was performed using the REDExtract-N-AmpTM Tissue PCR Kit (Sigma-Aldrich, Dorset, UK) according to manufacturer’s instructions. Primers to detect the TRPM7+/Δkinase (5’ tgc gag gcc aga ggc cac ttg tgt agc 3’; and 5’ tgc gag gcc aga ggc cac ttg tgt agc 3’) were designed to amplify the neighboring sequence regions of TRPM7-kinase domain. Therefore, the PCR product is only amplified in TRPM7+/Δkinase. For this reason, WT mice do not show any product amplification using these primers since the extension time in our PCR conditions do not allow amplification of such a large sequence (TRPM7 plus neighbor sequence). To confirm that the absence of PCR bands with these primers were truly WT mice and not a result obtained due to a poor DNA extraction and/or PCR performance, a control primer set (5’ aaa tct tag gct ggt aga cag tg 3’; and 5’ ctt atc tct caa gcc aat tta gga g 3’) independent of TRPM7+/Δkinase was generated to accurately interpret the data. Therefore, DNA amplification must be positive for WT and TRPM7+/Δkinase samples. The PCR protocol was as following: 1 cycle of 94 ^o^C (heat start), and 35 cycles of: 10 sec at 94 ^o^C (denaturation), 30 sec at 62 ^o^C (annealing), 2 min at 68 ^o^C (extension). One final cycle of 5 min at 68 ^o^C was performed, followed by temperature of 4 ^o^C. PCR products were visualized using agarose gel electrophoresis. A representative figure of the genotyping results is shown in Suppl. Fig. S1.

**1.2. Echocardiography**

Cardiac function and structure were assessed by echocardiography using an Acuson Sequoia c512 ultrasound system to acquire non-invasive 2-D guided M-mode images at a 20 mm depth at the tip of the papillary muscles. Measurements were made in a short axis view using the leading edge-to-lead edge convention during both systole and diastole over at least three consecutive cardiac cycles. Echocardiographic indices assessed included Left Ventricular Anterior Wall thickness (LVAW), LV End-Diastolic Volume (LVEDV), LV End-Systolic Volume (LVESV), LV End Diastolic Diameter (LVEDD), LV End-Systolic Diameter (LVESD), Fractional Shortening (FS) to assess left ventricular systolic function. Early (E) and late (atrial; A) ventricular filling velocity were calculated from measuring blood velocities across the mitral valve during each cardiac cycle. E/A ratio was calculated as an indirect measure of diastolic function. Ejection fraction (EF) = [(LVEDV – LVESV) / LVESD x 100]; Fractional shortening (FS) = [(LVEDD – LVESD) / LVEDD x 100].

**1.3. Plasma and urine biochemistry**

Blood was collected under isoflurane anesthesia (3% induction; 1.5% maintenance) by cardiac puncture immediately prior to sacrifice. Blood was collected in heparinized tubes (TekLab, County Durham, UK). Plasma was separated by centrifugation (2,000 rpm, 10 min) (Heraeus Megafuge 16R; ThermoScientific). Spot urine was collected from the bladder during sacrifice. Plasma and urine were aliquoted, snap frozen and stored at -80 ^o^C. Calcium, phosphate, sodium, potassium, chloride, magnesium, albumin, creatinine, plasma total cholesterol, HDL and glucose were determined by an automated analyzer (Roche/Hitachi cobas c systems - cobas c 311 Autoanalyser).

**1.4. Intravital microscopy to assess vascular inflammatory responses in vivo**

Venules were visualized using an intravital microscopy Leica M205FA, camera Hamamatsu digital C11440, using the software LAS X version 2.0.0.14332 (Leica Microsystems, Milton Keynes, UK). Vessels between 25 and 35 µm were randomly chosen from different areas of the tissue and videos were recorded for 60 sec. Leukocyte rolling velocity was analysed in at least 12 cells per venule using the plugin “manual tracking” on the software ImageJ 1.44p (Wayne Rasband, NIH, USA). The velocity was determined by the distance travelled (µm) divided by the time taken (sec) for each cell ^2^. Leukocyte adhesion was determined by counting the number of cells that were firmly attached to the vessel wall for at least 30 sec and normalized by vessel area. Leucocyte transmigration was determined by counting the number of cells that migrated to the tissues and normalized by area of the field.

**1.5. Flow Cytometry**

Kidneys and hearts were cut in small pieces ≤ 1 mm and digested in collagenase II (2 mg/mL) (Sigma-Aldrich, Dorset, UK) diluted in DMEM serum free with constant agitation at 37ºC for 40 min. Enzyme was inactivated by adding FBS 10% in final concentration. Digested tissues were filtered through a 70 µm cell strainer. Spleen cells were collected after mechanic dysruption and filtered through a 70 μm cell strainer. Cell suspension obtained from kidneys, hearts, and spleens were centrifuged at 300 x g for 10 min. The cell pellet was resuspended in ACK Lysis Buffer (NH_4_Cl 150 mM, KHCO_3_ 10 mM, Na_2_EDTA 0.1 mM, pH 7.2–7.4) for 3 min on ice to lyse erythrocytes, followed by addition of 40 mL of PBS and centrifugation at 300 x g for 10 min. Cells from kidneys and hearts were resuspended with 3 mL of PBS/FBS 2%, which was carefully added on top of 3 mL ficoll-hipaque (1,077 mg/mL) in order to isolate the mononuclear cells, according to manufacturer’s instructions. Total cell number was obtained by counting in a Neubauer chamber, using trypan blue 0.4% exclusion. Cells were resuspended in FACS buffer (PBS/FBS 2%) and stained with fluorescent-conjugated anti-mouse monoclonal antibodies for 30 min on ice and in the dark. Cells were washed twice in PBS and resuspended in 200 uL of FACS buffer. Cells from heart, kidney and spleen were labelled with cell surface markers using the following specific antibodies**:** anti-CD45-FITC (30-F11), anti-CD3-PE-Cy7 (145-2C11), anti-CD4-APC (GK1.5), anti-CD8-APC-Cy7 (53-6.7), anti-CD19-PerCP-Cy5.5 (6D5), anti-F4/80-Alexa-647 (BM8), anti-CD11c-PE-Cy7 (N418), anti-CD206-FITC (C068C2), anti-CD206-PE (C068C2), anti-CD11b-Alexa 647 (M1/70), anti-Ly-6C-APC/Cy7 (HK1.4) all from Biolegend (London, UK) and anti-CD45-PE (30-F11, BD Pharmingen, Oxford, UK). Expression of markers was analysed by fluorescence intensity using a FACS Canto II flow cytometer (BD Biosciences, Wokingham, UK). Results were analysed using FlowJo software (TreeStar, Ashland, USA).

**1.6. Mg^2+^ and Ca^2+^ concentration**

Dried tissues were weighed using an analytical balance, followed by digestion in nitric acid concentrated in a water bath at 65 ^o^C for 2 h with the lids closed. Samples were cleared by centrifugation at 10,000 x g for 1 min. Cleared samples were diluted 1/10 in milliQ H_2_O and further incubated in a water bath 65 ^o^C for 2 h with the lids closed, followed by centrifugation at 10,000 x g for 1 min. 5 µL of digested samples were transferred to microplates and the Mg^2+^ concentration was analysed by colorimetric reaction using the commercial kit Magnesium Gen.2 (Roche Diagnostics, Burgess Hill, UK). Absorbance at 600 nm was measured using a microplate reader Spectramax M (Molecular devices, Winnersh, UK). Concentration in mmol/L was obtained using a standard curve prepared with MgCl_2_ and normalized by dried weigh, as described ^3^.

Measurement of intracellular free Mg^2+^ concentration ([Mg^2+^]i) was assessed using magnesium green AM (Thermo Fisher Scientific, Life Technologies, Paisley, UK) This probe incorporates into cells and has a high affinity for Mg^2+^ (Kd ~1.0 mM). Upon binding to Mg^2+^, magnesium green exhibits an increase in fluorescence emission intensity without a shift in wavelength with absorption/emission maxima of ~506/531 nm. Briefly, isolated cells were resuspended in Ca^2+^ and Mg^2+^ free HEPES buffer (NaCl 150 mM, KCl 5 mM, D-glucose 10 mM, HEPES 20 mM) and labelled with magnesium green 5 μM for 30 min, at 37 ºC in the dark and in constant gently agitation to avoid cell adhesion. Cells were washed in PBS and then incubated in HEPES buffer at 37 ºC for additional 30 min to allow complete de-esterification of intracellular AM esters. Cells where then labelled with anti-F4/80-Alexa-647 (BM8), anti-CD206-FITC (C068C2), anti-CD11c-PE-Cy7 (N418), anti-CD11b-Alexa647 (M1/70), anti-Ly-6C-APC/Cy7 (HK1.4) all from Biolegend (London, UK). Magnesium green fluorescence was acquired using the FITC channel. Data acquisition was performed using Flow Cytometry (FACS Canto II, BD Biosciences, Wokingham, UK). Results were analyzed using FlowJo software (TreeStar, Ashland, USA).

To assess intracellular free Ca^2+^ concentration ([Ca^2+^]i), macrophages were loaded with 10 µM Cal-520 AM (Abcam, Cambridge, UK ) in RPMI/0.5% FBS at 37 °C for 75 minutes, followed by 30 minutes at room temperature. Cells were incubated in HEPES physiological saline solution (NaCl 130 mM, KCl 5mM, CaCl_2_ 1 mM, MgCl_2_ 1 mM , HEPES 20 mM, and D-glucose 10 mM, pH 7.4) for 30 minutes to allow de-esterification. Data acquisition was performed using Flow Cytometry (FACS Canto II, BD Biosciences, Wokingham, UK) using the FITC channel. Results were analyzed using FlowJo software (TreeStar, Ashland, USA).

**1.7. Culture of vascular smooth muscle cells (VSMC) from mice with high-normal (MgH) and low (MgL) levels of Mg^2+^**

Using bidirectional selective breeding, mice from a heterogeneous population were selected for high-normal (MgH) and low (MgL) levels of intracellular Mg^2+^ ^4^. MgL mice demonstrate inherited hypomagnesemia, with significant reductions in plasma, bone and kidney magnesium levels ^4^ ^5^. Mesenteric arteries were isolated and characterized as described in detail previously ^6^. Briefly, mesenteric beds were cleaned of adipose and connective tissue; VSMCs were dissociated by enzymatic digestion of vascular arcades for 60 minutes at 37°C. Cell suspension was centrifuged and resuspended in Dulbecco modified Eagle medium containing 10% fetal calf serum, 2 mmol/L glutamine, 20 mmol/L HEPES (pH 7.4), 100 U/ml penicillin G, and 100 mg/ml streptomycin, all from Gibco (Long Island, NY, USA). Cells in the passage P5-P7 were used in the experiments.

**1.8. Culture of bone marrow-derived macrophages**

Briefly, femurs from WT and TRPM7^+/Δkinase^ mice were flushed with cold PBS. Isolated bone-marrow cells were passed through a 26-G x 1/2” gauge needle and filtered in a 70 μm strainer. Cells were centrifuged at 400 x g for 10 min at 4 ºC and resuspended in RPMI supplemented with 2 mM l-glutamine, 100 U/ml penicillin G, and 100 mg/ml streptomycin, all from Gibco (Long Island, NY, USA), 20% LCM (L-929 cell conditioned medium) and 15% of heat-inactivated FBS. Cells were plated in cell culture plate P100 and incubated at 37 ºC in 5% CO_2_ (10 mL per plate). On day 3, an additional 10 mL of new fresh RPMI/15% FBS/ 20% LCM were added. On day 6, cells were detached from the plates using a cell scrapper, centrifuged and resuspended in RPMI/5% FBS. Total cell number was obtained by counting in a Neubauer chamber, using trypan blue 0.4% exclusion. BMDM were plated in 6-well plates at concentration 0.5 x 10^6^/mL in RPMI/5% FBS. After 2 h incubation, the medium was changed to RPMI/1% FBS. Plated cells were treated with MgCl_2_ 10 mM for 24 h. After this, cells were lysed and supernatants centrifuged (10,000 g for 10 min) and stored at -80 ºC in sterile conditions.

**1.9. Isolation of resident peritoneal macrophages**

Resident peritoneal macrophages were harvested from the peritoneal cavity of mice by lavage with cold PBS. Resident peritoneal were resuspended in RPMI 1640 (Life Technologies, Carlsbad, CA) containing 5% FBS (Gibco, Grand Island, NY) and allowed to adhere to tissue culture-treated plates for 2 h at 37 °C and 5% CO_2_. Cells were washed with pre-warmed PBS and cultured in RPMI containing 1% FBS (Invitrogen, Thermo Fisher, Paisley, UK). Cells were then stimulated with LPS (100 ng/mL) for 20 h.

**1.10. Culture of cardiac fibroblasts**

Hearts were cut into small pieces ≤ 1 mm and digested in 2 mL of collagenase II (2 mg/mL) (Sigma-Aldrich, Dorset, UK) diluted in DMEM serum free with constant agitation at 37ºC for 40 min. Enzymes were inactivated by adding 200 µL of FBS. Cell suspension was centrifuged at 400 g for 10 min. The resulting pellet was resuspended in 5 mL of DMEM/20% FBS transferred to a T-25 flask. After 16 h, the non-attached cells were removed by washing three times with warm PBS. Adherent cells were cultured in DMEM/20% and low passage cells (passage 3-6) were studied. For experiments, cardiac fibroblasts were plated in 6-well plate, until semiconfluent. Cells were maintained in DMEM/1% over-night, followed by treatment with TRPM7^+/Δkinase^ macrophage supernatant (diluted 50%) for 24 h.

**1.11 Macrophage-fibroblast co-cultures**

Cardiac fibroblasts were seeded in 6 well-tissue culture plates (Thermo Scientific, Darmstadt, Germany) DMEM/20% FBS until subconfluency and the medium was changed to 1.5mL of DMEM/1% FBS. Macrophages were differentiated from bone marrow cells in 10 mL of DMEM/10%FBS supplemented with 20 ng/mL recombinant murine M-CSF (Preprotech, London, UK) in a cell culture plate P100 and incubated at 37 ºC in 5% CO2 (10 mL per plate). On day 3, an additional 10 mL of new fresh DMEM/10% FBS and 20 ng/mL of M-CSF were added. On day 6, cells were detached from the plates using a cell scrapper, centrifuged and resuspended in DMEM/1% FBS. Differentiated macrophages were added on transwell inserts 0.4 µm at density 5x10^5^ cells/well in 0.5 mL of DMEM/5%. After 2 h, the non adherent cells were washed, medium was changed to 0.5 mL of DMEM/1% FBS and the inserts were added on top of the cell culture of cardiac fibroblasts for 48 h, followed by protein extraction.

**1.12. Immunoblotting**

Total protein from frozen tissues and cells was extracted in lysis buffer containing Tris 50 mM, pH 8.0, NaCl 150 mM, Triton X-100 1%, SDS 0.1%, supplemented with phenylmethylsulfonyl fluoride (PMSF) 1 mM, pepstatin A 1 µg/mL, leupeptin 1 µg/mL, aprotinin 1 µg/mL (Sigma-Aldrich, Dorset, UK), sodium fluorate 10 mM (AnalaR Normapur; VWR International, Leuven, Belgium), and sodium orthovanadate 1 mM (Alfa Aesar, Heysham, UK). Total protein lysate was sonicated, cleared by centrifugation at 10,000 rpm for 5 min and the pellet was discarded. Protein concentration was determined using the DC protein assay kit (Bio-Rad Laboratories, Watford, UK). Proteins (30 µg) were separated by electrophoresis on a polyacrylamide gel and transferred onto a nitrocellulose membrane (Thermo Scientific, Darmstadt, Germany). Nonspecific binding sites were blocked with 5% non-fatty dry milk solubilized in Tris-buffered saline solution with Tween 0.01 % for 1 hour at room temperature. Membranes were then incubated overnight at 4°C with the following primary specific antibodies: fibronectin (rabbit) α-smooth muscle actin (α-SMA), β-actin (mouse), (Sigma-Aldrich, Dorset, UK); phospho-Smad3 (mouse, S423 + S425, Abcam, Cambridge, UK), phospho-P66Shc (rabbit, Ser36, Calbiochem-Millipore, Watford, UK); TGFβ1, annexin-1, calpain-II, galectin-3, proliferating cell nuclear antigen (PCNA) (Rabbit, Santa Cruz, Heidelberg, Germany); spectrin αII (mouse C11, Santa Cruz, Heidelberg, Germany), α-tubulin, vimentin (rabbit), GAPDH (mouse, Abcam, Cambridge, UK), Na/K ATPase (rabbit) (Abcam, Cambridge, UK); phospho-Stat3 (Tyr705) (mouse), total-Stat3 (rabbit), phospho-Stat1 (Tyr701) (rabbit), total-Stat1 (mouse) (Cell Signaling Technology, Beverly, MA, USA), TRPM7 α-kinase domain (aa 1800-1900) (rabbit, EPR4582, Abcam, Cambridge, UK) and TRPM7 (aa 1817-1863) (mouse, S74-25, Invitrogen, Thermo Fisher, Paisley, UK) Next, membranes were washed with TBS-tween and incubated with secondary fluorescence-coupled antibodies goat-anti-mouse-IRDye 680 or goat-anti-rabbit-IRDye 800 (LI-COR, Cambridge, UK) 1 h, at room temperature in the dark and visualized by an infrared laser scanner (Odyssey Clx, LICOR, Cambridge, UK). Western blotting images were quantified using the software Image Studio™ Lite free version (LICOR, Cambridge, UK). Protein expression levels were normalized to loading controls and expressed as absolute values.

**1.13. Immunoprecipitation**

Total lysates of hearts tissues (400 µg) were incubated with the anti-TRPM7 (Abnova, Taoyuan City, Taiwan), specific to the amino acids 45-74 at N-terminus of TRPM7. Samples were incubated with the antibody under gentle agitation overnight at 4°C, followed by addition of 20 µL of protein A/G PLUS-Agarose (Santa Cruz, Heidelberg, Germany) and incubated for additional 4 h at 4 °C. Western blot was performed using the mouse anti-phosphoserine/threonine/tyrosine antibody (Abcam, Cambridge, UK). Protein expression was ocquired using the secondary fluorescence-coupled antibodies goat-anti-mouse-IRDye 680 (LI-COR, Cambridge, UK) in an infrared laser scanner (Odyssey Clx, LICOR, Cambridge, UK).

**1.14. Cytosol and membrane fractionation**

Translocation of annexin-1 and calpain-II from the cytosol to the membrane was assessed in cardiac tissues as a marker of TRPM7 kinase activity, since these proteins are downstream targets of TRPM7 kinase. Tissues were lysed in buffer A (Tris 50 mM, Na_2_EDTA 2 mM, PMSF 1 mM, leupeptin 1 μg/mL, aprotinin 1 μg/mL, and pepstatin 1 μg/mL) and ultracentrifuged at 100,000 x g for 1 h. The cytosolic fraction (supernatant) was collected and membrane fraction (pellet) was solubilized in buffer B (buffer A plus NaCl 300 mM, triton 100 1%, and SDS 0.1%). Western blotting from cytosol and membrane enriched fractions was performed as described using anti-annexin-1, anti-calpain-II antibodies (Santa Cruz Biotechnology, Inc). Na-K ATPase and α-tubulin were used as loading control for the membrane and cytosolic fractions respectively. Translocation was determined as the ratio of protein expression in membrane to cytosolic fractions.

**1.15. Real-Time Reverse-Transcription Polymerase Chain Reaction.**

Total RNA was isolated using the QIAzol Lysis Reagent (Qiagen, Manchester, UK) according to the manufacturer’s instructions and diluted in nuclease-free H_2_O (Ambion/Life Technologies, Paisley, UK). cDNA was generated from total RNA using the High-Capacity cDNA Reverse Transcription Kits (Applied Biosystems, Warrington, UK). Real-time polymerase chain reaction was performed with the Applied Biosystems 7900HT Fast Real-Time PCR system, using Power SyBr Green Master Mix (Applied Biosystems, Warrington, UK) and specific murine primers to GAPDH, fibronectin, collagen-1, TGFβ1, TNFα, IL-1β, IL-12, IL-10, Arg1, iNOS, IFN-γ, VCAM-1, MMP2, TIMP-1, all acquired from Eurofins genomics (Glasgow, UK) (Supplemental Table 1). Relative gene expression was calculated by the 2^-∆∆Ct^ cycle threshold method as previously described ^7^.

**1.16. Histology**

Hearts, aortas, and kidneys were fixed in 10% buffered-formalin solution and processed for histological inclusion in paraffin. Five-µm thick tissue sections were stained with Haematoxylin and Eosin (HE) and PicroSirius red for light microscopy. Fibrosis was further analysed in PicroSirius red stained samples using polarized light microscopy. 15 randomly selected non-overlapping fields (200×) were analysed using the package ImageJ 1.44p (Wayne Rasband, NIH, USA) available in <http://imagej.nih.gov/ij>. Tissue fibrosis was also quantified using second harmonic generation/two photon excitation fluorescence (SHG/TPEF) in a Genesis system (HistoIndex, Singapore). Image acquisition was performed with constant parameters for all samples, using a 20× objective on unstained sections. SHG/TPEF analysis was performed by HistoIndex software (HistoIndex, Singapore).

**1.17. Immunohistochemistry**

Mouse hearts sections (5 µm) were deparaffinized in Histoclear (Biocity Scotland, UK) hydrated in ethanol, and treated in citrate buffer (citric acid 0.1 M, sodium citrate 0.1 M, Tris 0.2 M) pH 7.2, 98 ºC, for 40 minutes, for the antigen retrieval. Slides were blocked with TBS-Tween 20 (0.2%) - Casein (0.06%) (TBS-TC) for 10 min and incubated, overnight at 4 ºC, with the primary antibody anti-galectin 3 (1:400) (Rabbit, Santa Cruz, Heidelberg, Germany). Slides were washed in PBS and TBS-TC for 5 min, and incubated with avidin-biotin-peroxidase complex (ABC kit; Vector Laboratories, Peterborough, UK) according to the manufacture’s instruction. The slides were incubated with Diaminobenzidine chromogen (DAB kit, Vector Laboratories, Peterborough, UK), rinsed and counterstained with haematoxylin, de-hydrated and mounted with a synthetic medium. To exclude antigen-independent staining, the primary antibody was replaced by nonspecific rabbit IgG (isotype control).

**1.18. ELISA**

Plasma galectin-3 levels were analysed by ELISA (Thermo Scientific-Pierce, Paisley, UK). Cytokine production was analysed in the macrophage supernantant by ELISA: Mouse IL-10 and Mouse IL-12p70 ELISA Ready-SET-Go! (Invitrogen, Thermo Fisher, Paisley, UK), Mouse IL-6 and Mouse TNF-α Antibody Pair (Life Technologies, Thermo Fisher, Paisley, UK).

**Supplemental references**

1. Ryazanova LV, Rondon LJ, Zierler S, Hu Z, Galli J, Yamaguchi TP, Mazur A, Fleig A, Ryazanov AG. TRPM7 is essential for Mg(2+) homeostasis in mammals. *Nature communications* 2010;**1**:109.

2. Megens RT, Soehnlein O. Intravital Microscopy for Atherosclerosis Research. *Methods in molecular biology* 2015;**1339**:41-60.

3. Arjona FJ, de Baaij JH, Schlingmann KP, Lameris AL, van Wijk E, Flik G, Regele S, Korenke GC, Neophytou B, Rust S, Reintjes N, Konrad M, Bindels RJ, Hoenderop JG. CNNM2 mutations cause impaired brain development and seizures in patients with hypomagnesemia. *PLoS genetics* 2014;**10**:e1004267.

4. Henrotte JG, Franck G, Santarromana M, Frances H, Mouton D, Motta R. Mice selected for low and high blood magnesium levels: a new model for stress studies. *Physiol Behav* 1997;**61**:653-658.

5. Yogi A, Callera GE, O'Connor SE, He Y, Correa JW, Tostes RC, Mazur A, Touyz RM. Dysregulation of renal transient receptor potential melastatin 6/7 but not paracellin-1 in aldosterone-induced hypertension and kidney damage in a model of hereditary hypomagnesemia. *J Hypertens* 2011;**29**:1400-1410.

6. Montezano AC, Lopes RA, Neves KB, Rios F, Touyz RM. Isolation and Culture of Vascular Smooth Muscle Cells from Small and Large Vessels. *Methods Mol Biol* 2017;**1527**:349-354.

7. Livak KJ, Schmittgen TD. Analysis of relative gene expression data using real-time quantitative PCR and the 2(-Delta Delta C(T)) Method. *Methods* 2001;**25**:402-408.

**Supplemental table 1**. Primer sequences

|  | Forward 5’-3’ | Reverse 5’-3’ |
| --- | --- | --- |
| TRPM7 | TTTGGTGTTCCCAGAAAAGC | ACCAAGTTCCAGGACCACAG |
| TRPM6 | CCTCACGGCTCTACTGAAGG | ACCAGGCTTCCAATGTTGTC |
| Fibronectin | CCGGTGGCTGTCAGTCAGA | CCGTTCCCACTGCTGATTTATC |
| Col1a1 | GAGCGGAGAGTACTGGATCG | GACCTCGTGCTCCAGTTAGC |
| TGFβ1 | GTCCTTGCCCTCTACAACCA | GTTGGACAACTGCTCCACCT |
| IL-1β | GCCTCGTGCTGTCGGACCCATAT | TCCTTTGAGGCCCAAGGCCACA |
| IL-12 | TGGTTTGCCATCGTTTTGCTG | ACAGGTGAGGTTCACTGTTTCT |
| IL-10 | CAGAGCCACATGCTCCTAGA | TGTCCAGCTGGTCCTTTGTT |
| Arg1 | TTCTCAAAAGGACAGCCTCG | AGCTCTTCATTGGCTTTCCC |
| MCP-1 | CCCACTCACCTGCTGCTACT | TCTGGACCCATTCCTTCTTG |
| iNOS | AATCTTGGAGCGAGTTGTGG | CAGGAAGTAGGTGAGGGCTTG |
| TNFα | CATCTTCTCAAAACTCGAGTGACAA | TGGGAGTAGATAAGGTACAGCCC |
| IFN-γ | GCGTCATTGAATCACACCTG | TGAGCTCATTGAATGCTTGG |
| VCAM-1 | ATTTTCTGGGGCAGGAAGTT | ACGTCAGAACAACCGAATCC |
| MMP2 | ACACTGGGACCTGTCACTCC | TGTCACTGTCCGCCAAATAA |
| TIMP-1 | CATGGAAAGCCTCTGTGGAT | CTCAGAGTACGCCAGGGAAC |
| GAPDH | AGGTCGGTGTGAACGGATTTG | TGTAGACCATGTAGTTGAGGTCA |

**Supplemental Table 2.** Plasma and Urine analysis

|  | WT | TRPM7^+/Δkinase^ |
| --- | --- | --- |
| *Plasma* |  |  |
| Cholesterol | 1.17 ± 0.18 | 1.50 ± 0.06 * |
| Triglycerides | 1.08 ± 0.09 | 1.24 ± 0.06 |
| HDL | 1.74 ± 0.05 | 1.58 ± 0.04 * |
| Glucose | 11.84 ± 0.69 | 12.77 ± 0.35 |
| Phosphate | 1.93 ± 0.04 | 1.87 ± 0.01 |
| Potassium | 5.38 ± 0.69 | 4.78 ± 0.14 |
| Magnesium | 0.84 ± 0.04 | 0.76 ± 0.04 |
| Calcium | 2.28 ± 0.05 | 2.32 ± 0.02 |
| Sodium | 146.5 ± 0.76 | 146.4 ± 0.53 |
| Chloride | 31.60 ± 0.08 | 31.11 ± 0.11 |
| Galectin-3 | 1470 ± 220.3 | 2533 ± 278.0 * |
|  |  |  |
| *Urine* |  |  |
| Albumin | 57.46 ± 16.1 | 66.37 ± 19.1 |
| Phosphate | 152.10 ± 48.8 | 31.90 ± 10.3 * |
| Potassium | 175.90 ± 36.1 | 100.6 ± 16.0 * |
| Magnesium | 14.73 ± 3.19 | 6.45 ± 0.85 * |
| Calcium | 5.13 ± 1.71 | 2.36 ± 0.39 |
| Sodium | 844.4 ± 343.0 | 567.8 ± 178.9 |
| Chloride | 1253 ± 375.5 | 504.3 ± 149.0 * |

Plasma parameters for cholesterol, triglycerides, HDL, and electrolytes are expressed in mmol/L (n=14/group) . Plasma galectin-3 is expressed in pg/mL (n=6/group). Urine electrolytes is expressed as mmol/L and normalized by creatinine concentration (mmol/L). Urine albumin is expressed as mg/dL and normalized by creatinine concentration (mmol/L) (WT n=8, TRPM7^+/Δkinase^ n= 10) .

**Supplemental Figure Legends**

**Supplemental Figure S1. Genotyping strategy to observe the mutation in TRPM7^+/Δkinase^ mice.** DNA isolated from mouse ear biopsies of 3 weeks old animals were analyzed by PCR to confirm the presence of an introduced Neo-cassette (red arrow). Lanes 1, 2, 3, 6 and 9 represent PCR products amplified from TRPM7^+/Δkinase^ animals and Lanes 4, 5, 7 and 5 representative DNA samples of WT (TRPM7^+/+^) animals.

**Supplemental Figure S2. Intracellular Mg^2+^ in macrophages and TRPM6 and TRPM7 expression in hearts and kidneys.** Total mononuclear leukocytes were isolated from (A) hearts (n=5) and (B) kidneys (WT n=5, M7+/Δ n=6), and labelled with Magnesium green AM, followed by specific antibodies anti-F4/80-Alexa647, anti-CD11c-PE-Cy7 for M1-macrophages, anti-206-PE for M2-macrophages. Scatter-plot graphs show mean of fluorescence intensity (MFI) according to free Mg^2+^ levels in different macrophage population. (C-F) Total RNA was extracted from hearts and kidneys and gene expression for (C-D) TRPM7 and (E-F) TRPM6 was analyzed by real time PCR and normalized to GAPDH (WT n=9, M7+/Δ n=10). PCR data are expressed in 2^-ΔΔCt^. Data are presented as mean ± SEM. Statistical significance was determined by a two-tailed unpaired Student’s t-test *P<0.05 TRPM7^+/Δkinase^ (M7+/Δ, blue) vs WT (yellow).

**Supplemental Figure S3. Expression and phosphorylation of TRPM7.** Total tissue lysates were investigated for the expression of TRPM7-kinase (A) in hearts using the mouse anti-TRPM7 (aa 1817-1863 at C-terminus, kinase domain, n=7) and (B) in kidneys using rabbit anti-TRPM7 (aa 1800-1900 at C-terminus, kinase domain, WT n=9, M7+/Δ n=11) and normalized by α-tubulin. (C) Total fresh lysates from hearts (400 µg total protein, n=5) were immunoprecipitated using rabbit anti-TRPM7 (aa 45-74 at N-terminus), followed by immunoblotting using mouse anti-phospho-Ser/Thr/Tyr and rabbit anti-TRPM7 (aa 45-74). (D) Total tissues from hearts (n=5) were investigated for the expression of TRPM7 channel using rabbit anti-TRPM7 (aa 45-74) and normalized by α-tubulin. Images were obtained using fluorescent secondary anti-mouse-IRDye 680 or anti-rabbit-IRDye 800 in a LI-COR system. Statistical significance was determined by a two-tailed unpaired Student’s t-test *P<0.05 TRPM7^+/Δkinase^ (M7+/Δ, blue) vs WT (yellow).

**Supplemental Figure S4. Cardiac fibrosis in TRPM7^+/Δkinase^ mice**. Cardiac sections from WT and TRPM7^+/Δkinase^ (M7+/Δ) mice were stained with sirius red. Collagen content was assessed using polarized light and analyzed for (A) mature collagen (red fluorescence), and (B) immature collagen (green fluorescence) (n=6/group). Representative images for polarized light microscopy are shown in the main figure 2H-lower panel. (C) Images were obtained by second harmonic generation (SHG), scale bars = 500 μm. Total RNA was extracted from hearts, and gene expression for (D) TGFβ1, (E) collagen-1 (Col1a1), and (F) fibronectin was analyzed by real time PCR and normalized to GAPDH (WT n=9, M7+/Δ n=10). PCR data are expressed in 2^-ΔΔCt^. (G) Cardiac expression of phospho p66Sch (Ser36) was observed by immunoblotting and normalized by α-tubulin (n=6/group). Images are representative of the experiment and data are presented as mean ± SEM. Statistical significance was determined by a two-tailed unpaired Student’s t-test. *P<0.05 TRPM7^+/Δkinase^ (M7+/Δ, blue) vs WT (yellow).

**Supplemental Figure S5. Immune cell infiltrate in cardiac tissues.**

Total mononuclear cells were isolated from hearts (n=5/group) and labeled for different cell populations: (A) CD45+CD3+ T lymphocytes; (B) CD45+CD3+CD8+ T lymphocytes (CD8+ T cells); (C) CD45+CD3+CD4+ T lymphocytes (CD4+ T cells); (D) CD45+CD19+ B cells (CD19+ cells). Data are presented as mean ± SEM. Statistical significance was determined by a two-tailed unpaired Student’s t-test. *P<0.05 TRPM7^+/Δkinase^ (M7+/Δ, blue) vs WT (yellow).

**Supplemental Figure S6. Collagen content and pro-fibrotic markers in vascular sections from WT and TRPM7^+/Δkinase^ mice.** (A-B) Histological sections from aortas of WT and TRPM7^+/Δkinase^ (M7+/Δ) were stained with picrosirius red and the collagen content was assessed using bright field microscopy. Scale bars = 10 μm. Images were analyzed and data expressed in % affected area (WT n=7, M7+/Δ n=6). (C) Images were obtained by second harmonic generation (SHG). Total RNA was obtained from aortas and gene expression was determined by real-time PCR for (D) Collagen-1, (E) Fibronectin, and (F) TGFβ1. (G) TIMP1 and MMP2 gene expression were analyzed by PCR and data expressed as TIMP1 to MMP2 ratio. PCR data were normalized by GAPDH and results are expressed in values of 2^-ΔΔCt^ (n=7/group). Graph data are presented as mean ± SEM. Statistical analyses was performed using a two-tailed unpaired Student’s t-test. TRPM7^+/Δkinase^ (M7+/Δ, blue) vs WT (yellow).

**Supplemental Figure S7~~.~~Pro-inflammatory and pro-fibrotic markers in kidneys from WT and TRPM7^+/Δkinase^ mice.**  (A-B) Total leukocytes were isolated from kidneys by enzymatic digestion and stained for flow cytometry analysis and B cells were defined as CD45+CD19+ cells. Results are shown as representative plots obtained from WT and TRPM7^+/Δkinase^  (M7+/Δ) samples and as mean ± SEM (n=5/group). Total RNA was obtained from kidney samples and gene expression was determined by real-time PCR for (C) TNFα, (D) IFNγ, (E) IL-1β, (F) Collagen-1, (G) TGFβ1, and (H) Fibronectin. Gene expression was normalized by GAPDH and PCR results are expressed in values of 2^-ΔΔCt^ (WT n=7, M7+/Δ n=9). Total tissue lysates were obtained from kidneys and investigated for the expression of (I) Fibronectin and (J) TGFβ1 by western-blotting and normalized by α-tubulin (α-tub) that was used as a loading control. Graph data are presented as mean ± SEM (n=6/group); Statistical significance was determined by a two-tailed unpaired Student’s t-test. *P<0.05 TRPM7^+/Δkinase^ (M7+/Δ, blue) vs WT (yellow). Abbreviations: SSC-A, Side-Scatter.

**Supplemental Figure S8.** **Spleens from TRPM7^+/Δkinase^ mice have increased lymphocytes, macrophages and fibrotic markers.** (A) Representative examples of spleens isolated from WT (left) and TRPM7^+/Δkinase^ (M7+/Δ) (right). (B) Weight was measured in freshly isolated spleens (mg) and normalized by the tibia length (cm) (WT n=10, M7+/Δ n=13). Total cells were isolated from spleens (n=5-6/group) and labeled for different cell populations: (C) Total hematopoietic immune cells (CD45+); (D) CD45+CD3+CD4+ T lymphocytes (CD4+ T cells); (E) CD45+CD3+CD8+ T lymphocytes (CD8+ T cells); (F) total macrophages (CD45+F4/80+ cells); (G) CD45+F4/80+CD11c+ cells (M1 macrophages) and CD45+F4/80+CD206+ cells (M2 macrophages) and data are expressed as ratio of the frequencies of CD11c+ to CD206+ macrophages; (H) CD45+CD19+ cells (B cells). Total RNA was purified from spleens samples and gene expression was determined by real-time PCR for (I) IFNγ, (L) IL-6, and (K) IL-10. Gene expression was normalized by GAPDH and PCR data are expressed in values of 2^-ΔΔCt^ (n=7/group). Total lysates obtained from spleens were investigated for the protein expression of (L) fibronectin and (M) TGFβ1 by western-blotting and normalized by α-tubulin (α-tub) that was used as a loading control (n=5/group). Graph data are presented as mean ± SEM; Statistical significance was determined by a two-tailed unpaired Student’s t-test. *P<0.05 TRPM7^+/Δkinase^ (M7+/Δ, blue) vs WT (yellow).

**Supplemental Figure S9.** **Blood monocytes from WT and TRPM7^+/Δkinase^ mice exhibit similar activation phenotype.** Mononuclear cells were isolated by density gradient centrifugation (1.077 g/mL). Monocytes were identified by flow cytometry as (A) CD45+CD11b+Ly6C+ total monocytes, (B) CD45+CD11b+Ly6C+^high^ inflammatory monocytes, and (C) CD45+CD11b+Ly6C+^low^ tissue repair monocytes (n=6/group). Results are shown as mean ± SEM. Statistical significance was determined by a two-tailed unpaired Student’s t-test. *P<0.05 TRPM7^+/Δkinase^ (M7+/Δ, blue) vs WT (yellow).

**Supplemental Figure S10. Macrophages from TRPM7^+/Δkinase^ exhibited increased calpain II expression and reduced intracellular Mg^2+^.**

Macrophages were differentiated from bone marrow isolated from WT and TRPM7^+/Δkinase^ (M7+/Δ) and assessed for (A) annexin-1 (n=8/group) and (B) calpain-II (n=6/group) by western blotting and normalized by loading control GAPDH. Macrophages were treated with MgCl_2_ (10 mM) for 24 h. (C) Intracellular concentration of Mg^2+^ was investigated using magnesium green AM (Mg^2+^ green) and fluorescence intensity (MFI) was analysed by FACS. (D) IL-6 production was investigated in the supernatant by ELISA. In (A-B) statistical significance was determined by a two-tailed unpaired Student’s t-test. In (C-D) statistical significance was determined by one-way ANOVA using the Student-Newman-Keuls post-test. TRPM7^+/Δkinase^ (M7+/Δ, blue), WT (yellow).*P<0.05 TRPM7^+/Δkinase^ (M7+/Δ) vs WT. **†** TRPM7^+/Δkinase^ treated with MgCl_2_ vs untreated TRPM7^+/Δkinase^.

**Supplemental Figure S11. Increased cytokine production by resident peritoneal macrophages from TRPM7^+/Δkinase^ mice.** Resident peritoneal macrophages were obtained by peritoneal lavage from WT and TRPM7^+/Δkinase^ (M7+/Δ) mice and stimulated with LPS (100 ng/mL) for 16 h. Cell supernatant was collected and the concentration of (A) IL-10, (B) IL-12p70, (C) IL-6 and (D) TNFα (n=6/group) was determined by ELISA. Cytokine values were normalized by total RNA (ng/mL). Results are shown as mean ± SEM. Statistical significance was determined by one-way ANOVA using the Student-Newman-Keuls post-test. *P<0.05 for TRPM7+/Δkinase (blue) vs WT mice (yellow). **†** LPS treated TRPM7^+/Δkinase^ vs non-treated TRPM7^+/Δkinase^. **‡** LPS treated TRPM7^+/Δkinase^ vs LPS treated WT.

**Supplemental Figure S12. Expression of vimentin and α-SMA in cardiac fibroblasts.** Primary culture of cardiac fibroblasts from WT mice were co-cultured in transwell system with macrophage from M7+/Δ and WT animals, treated or not with MgCl_2_. After 48 h co-culture, the total cell lysate was obtained from cardiac fibroblasts and expression of (A) α-SMA and (B) Vimentin (n=8/group) was analyzed by western blotting and normalized to β-actin. Data are presented as representative figures and mean ± SEM of TRPM7^+/Δkinase^ (M7+/Δ, blue) vs WT (yellow). Statistical significance was determined by one-way ANOVA using the Student-Newman-Keuls post-test.

**Supplemental Figure S13. Supernantants of TRPM7^+/Δkinase^ macrophages induce a fibrotic phenotype in cardiac fibroblasts from wild-type (WT) mice: effects of MgCl_2_ treatment**. (A) Macrophages were differentiated from bone marrow isolated from WT and TRPM7^+/Δkinase^ (M7+/Δ) mice. Primary culture cardiac fibroblasts from WT mice were stimulated with TRPM7^+/Δkinase^ macrophage supernatant treated or not with MgCl_2_. After 24 h stimulation, the total cell lysate was obtained from cardiac fibroblasts and the expression of (B) Fibronectin, (C) PCNA, (D) Vimentin, and (E) α-SMA was analyzed by western blotting and normalized by β-actin. Data are presented as representative figures and mean ± SEM (n=5/group). Statistical analysis performed using the one-way ANOVA using the Student-Newman-Keuls post-test of TRPM7^+/Δkinase^ (M7+/Δ, blue) vs WT (yellow). *P<0.05 for TRPM7^+/Δkinase^ (M7+/Δ) compared with WT mice. **†** TRPM7^+/Δkinase^ treated with MgCl_2_ vs untreated TRPM7^+/Δkinase^.

**Supplemental Figure S14. Expression of (A) fibronectin and (B) TGFβ in vascular smooth muscle cells from MgH and MgL mice.** Results are normalized to α-tubulin (n=6/group). Data are presented as mean ± SEM of MgH (yellow) vs MgL(blue). Statistical significance was determined by a two-tailed unpaired Student’s t-test *P<0.05 vs MgH
