# Supplementary figures and images for: Chanzyme TRPM7 protects against cardiovascular inflammation and fibrosis
Source: Cardiovasc Res. 2019 Jun 28;116(3):721–35. doi: 10.1093/cvr/cvz164 (PMC7252442; doi:10.1093/cvr/cvz164)

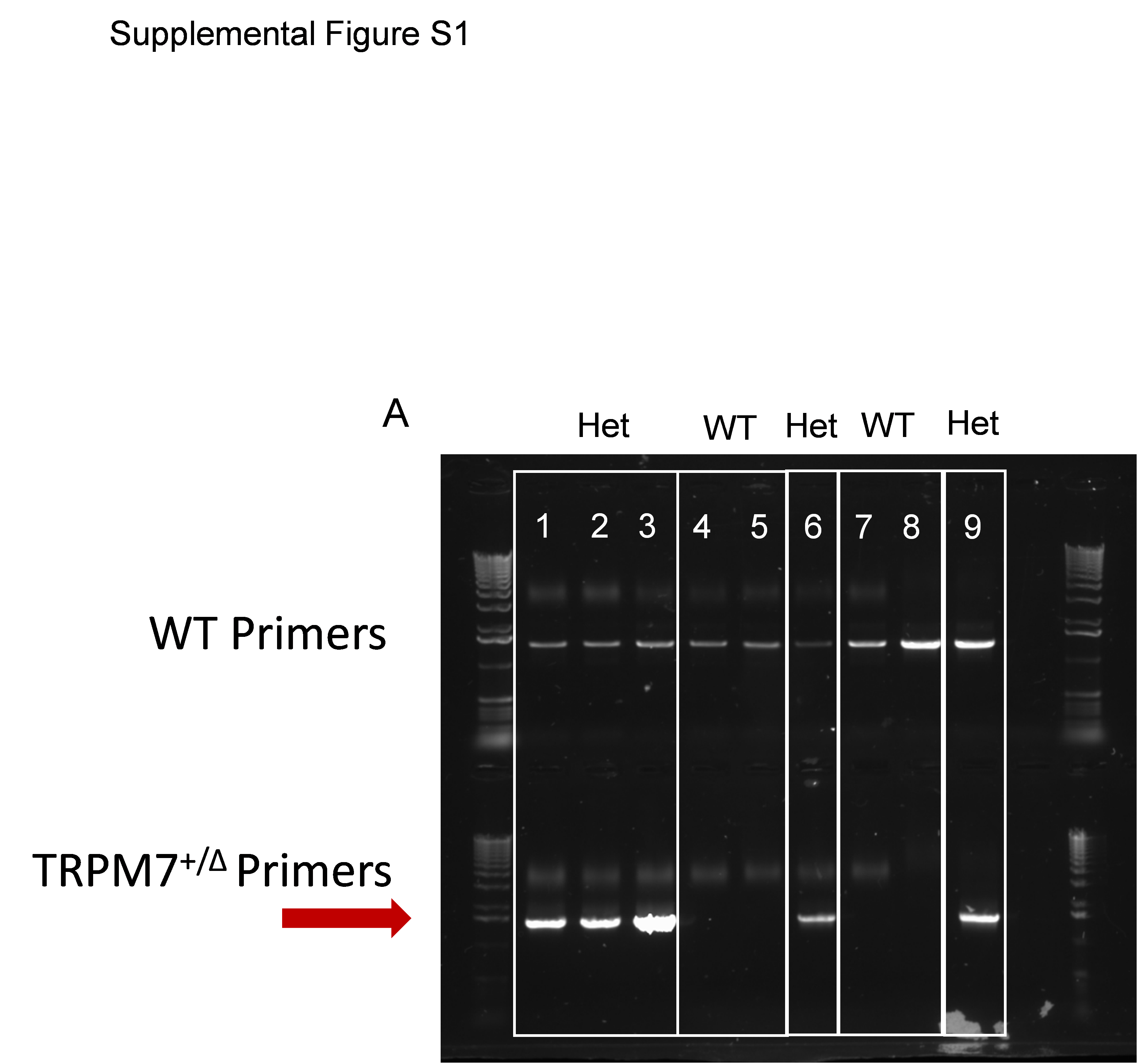

Supplement: cvz164_Supplementary_Data [file cvz164_supplementary_data.zip › cvz164-suppl_data/Supp Figure 1.tif]

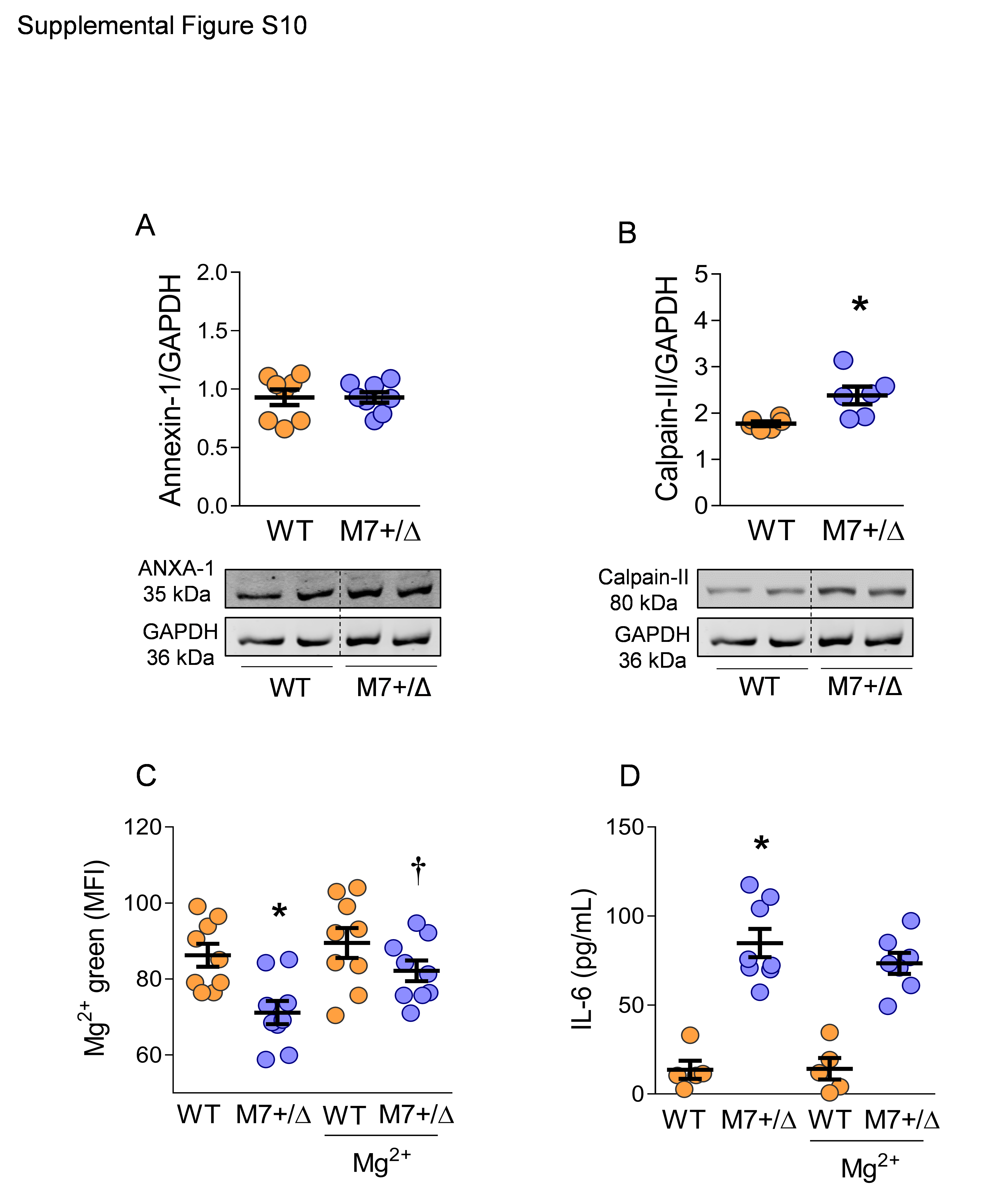

Supplement: cvz164_Supplementary_Data [file cvz164_supplementary_data.zip › cvz164-suppl_data/Supp Figure 10.tif]

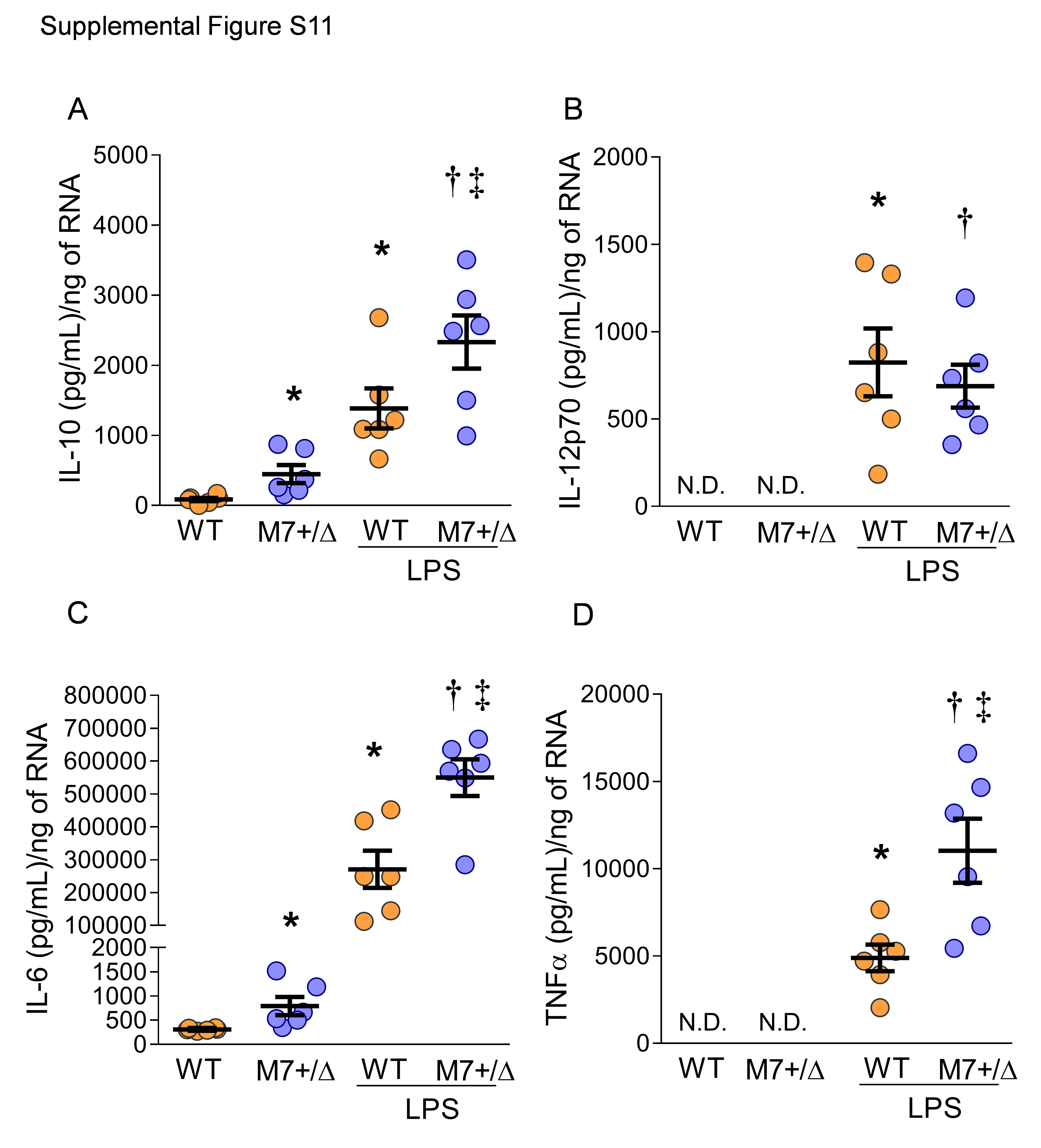

Supplement: cvz164_Supplementary_Data [file cvz164_supplementary_data.zip › cvz164-suppl_data/Supp Figure 11.tif]

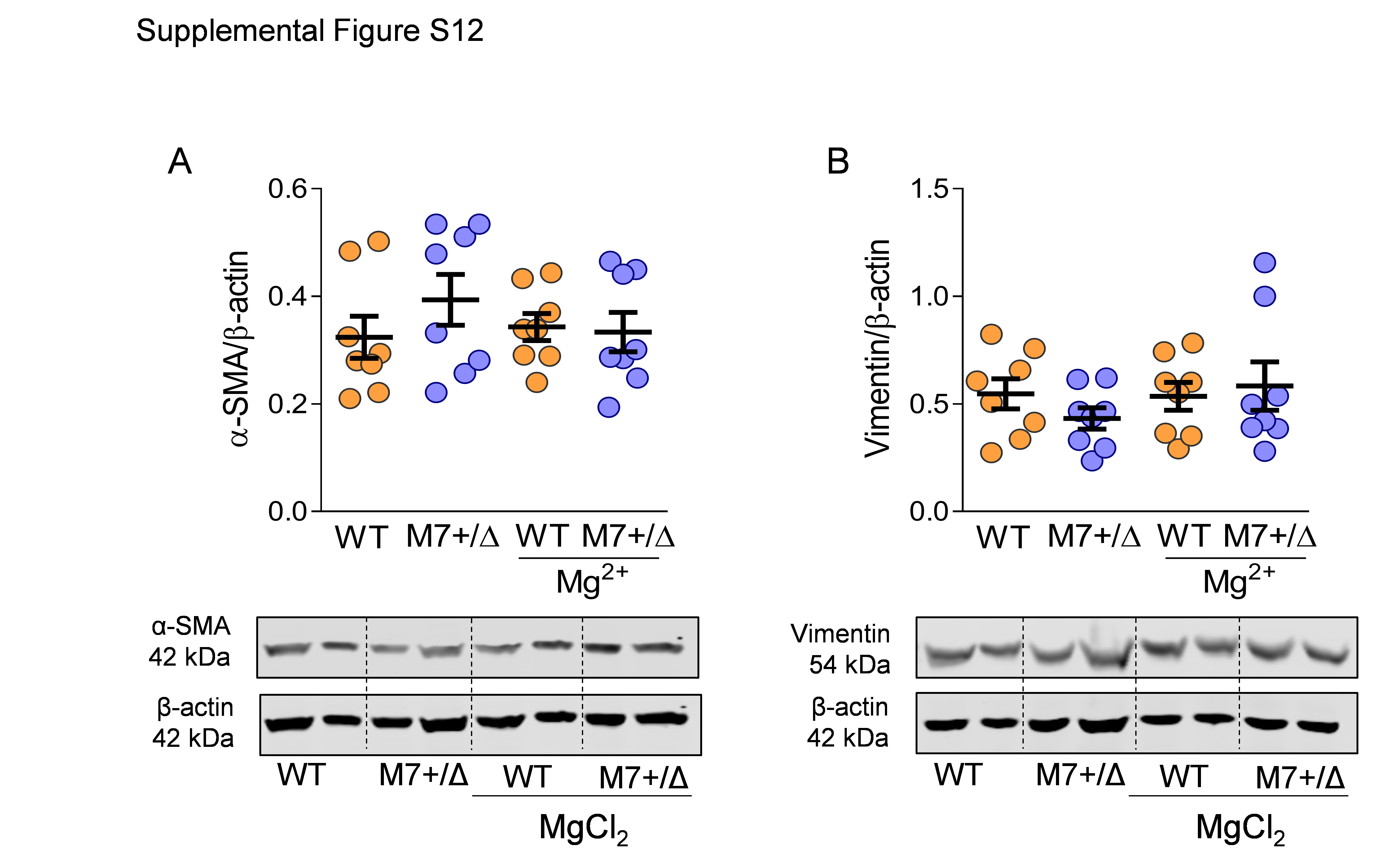

Supplement: cvz164_Supplementary_Data [file cvz164_supplementary_data.zip › cvz164-suppl_data/Supp Figure 12.tif]

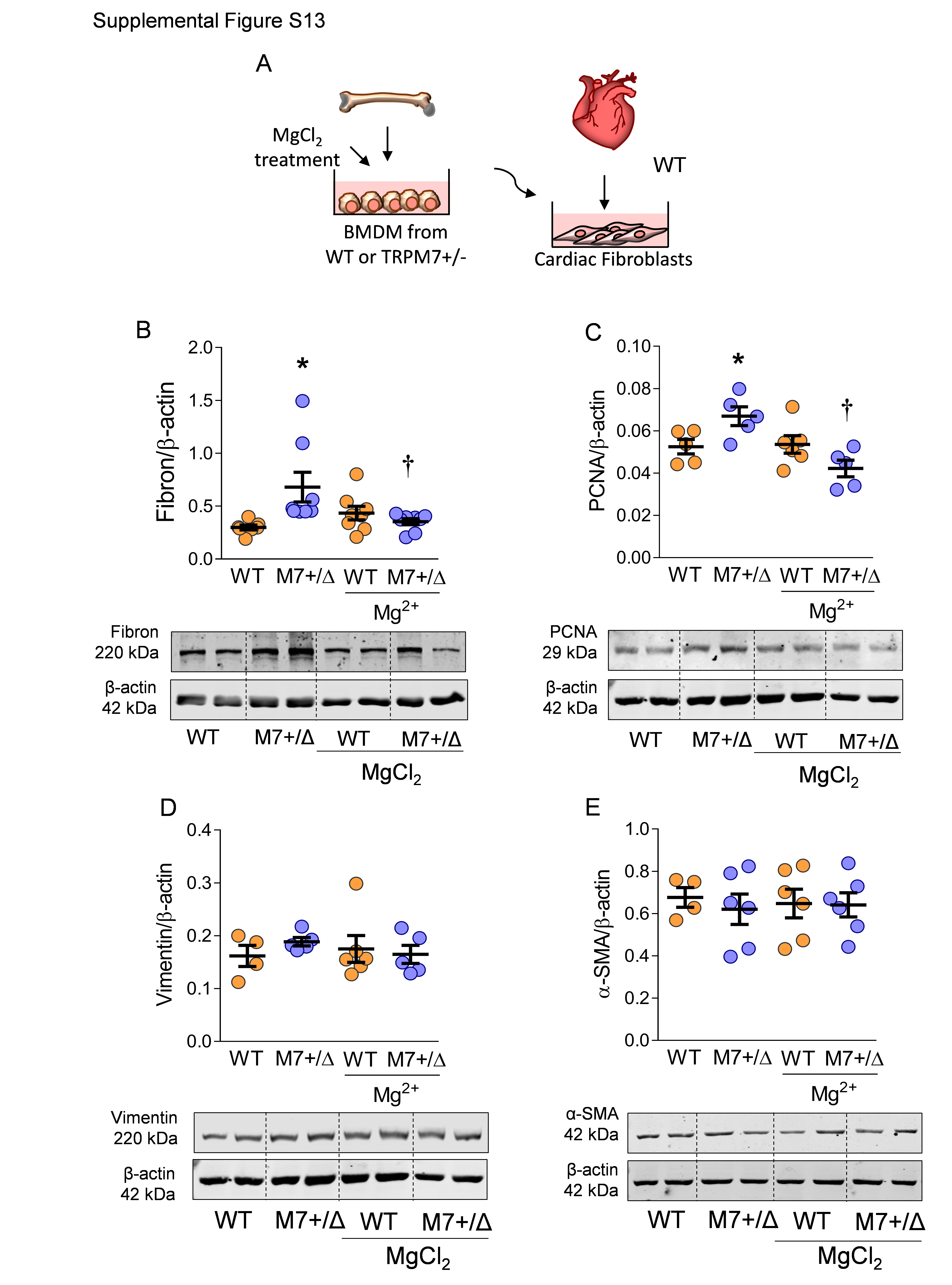

Supplement: cvz164_Supplementary_Data [file cvz164_supplementary_data.zip › cvz164-suppl_data/Supp Figure 13.tif]

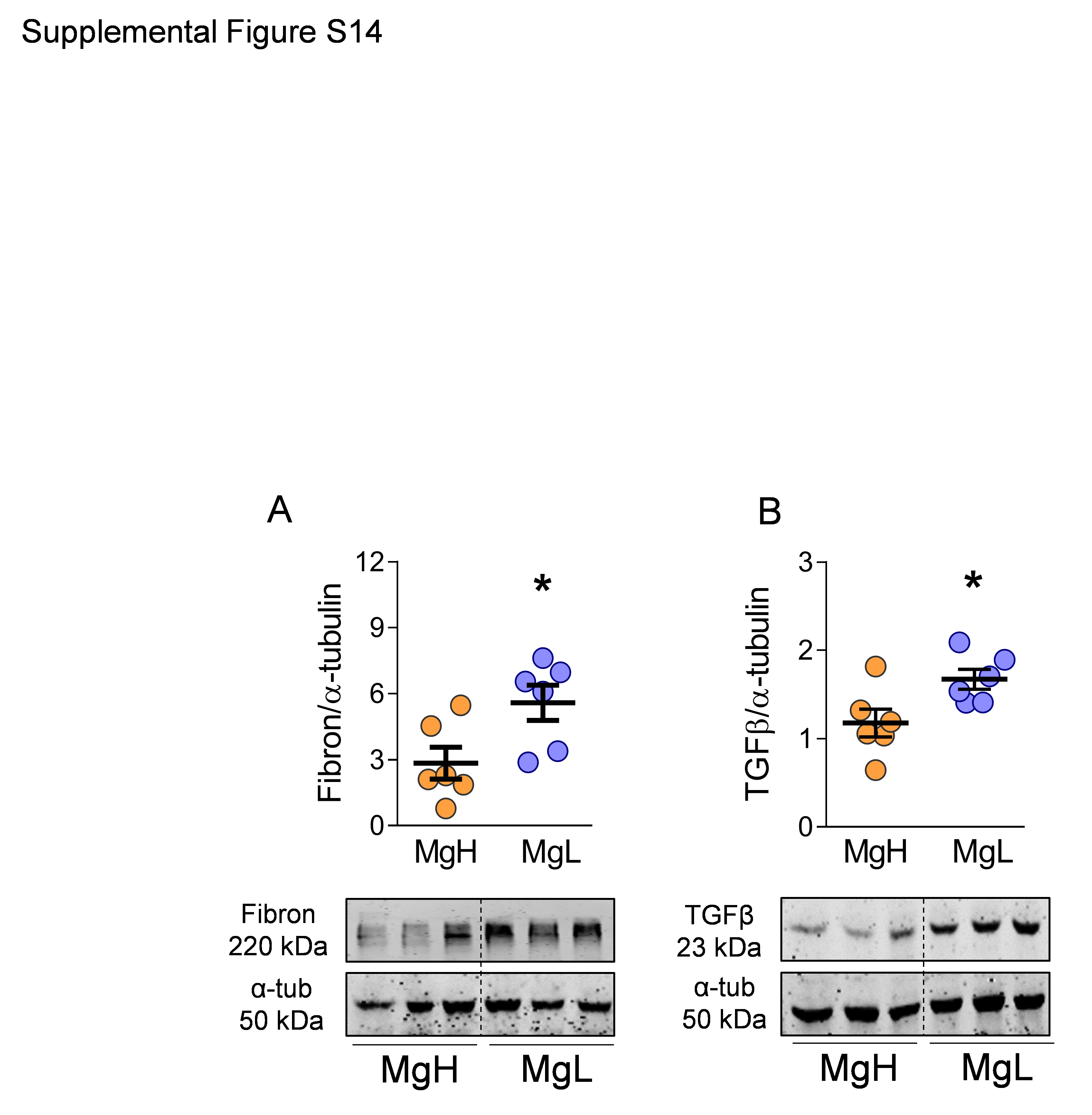

Supplement: cvz164_Supplementary_Data [file cvz164_supplementary_data.zip › cvz164-suppl_data/Supp Figure 14.tif]

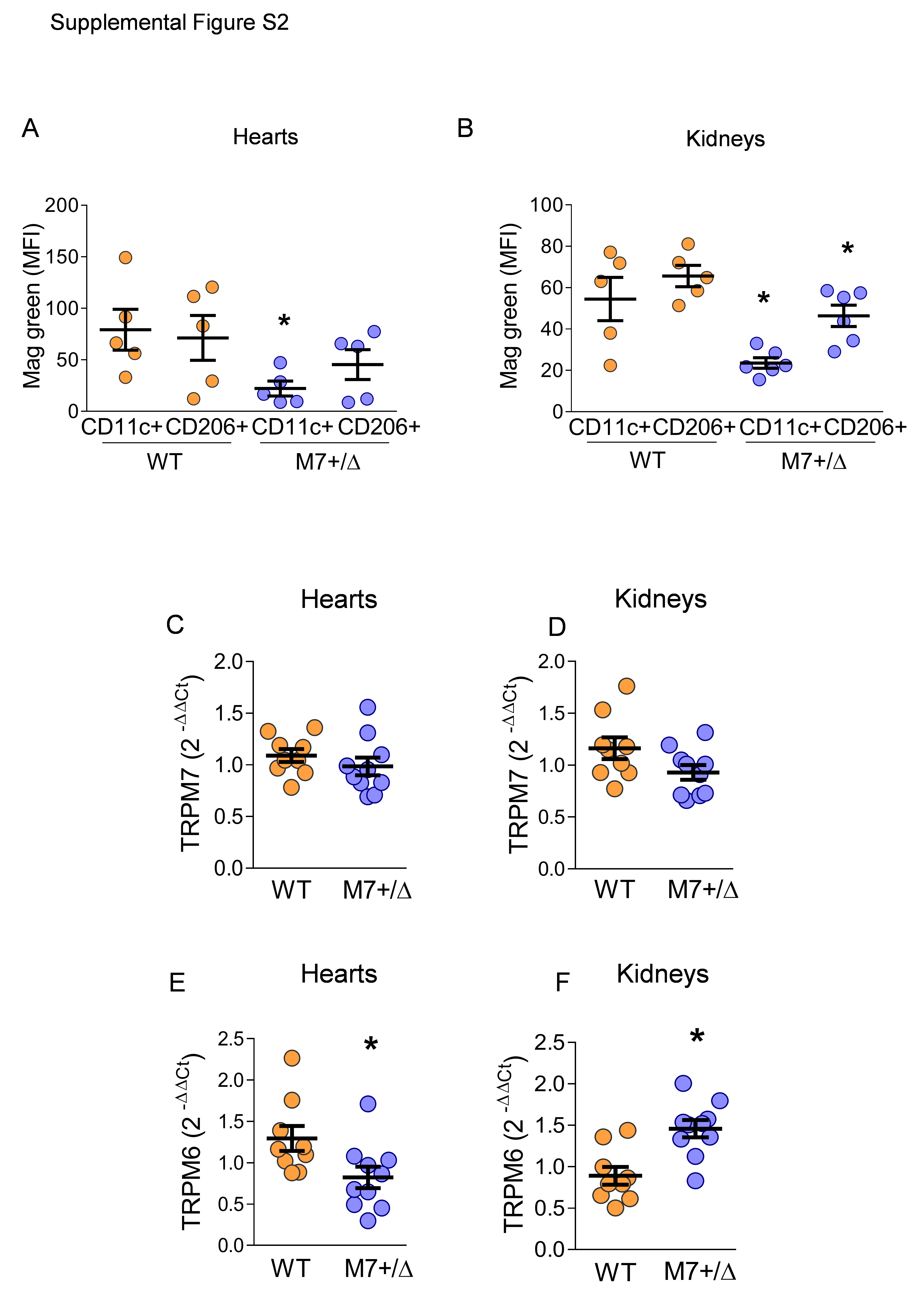

Supplement: cvz164_Supplementary_Data [file cvz164_supplementary_data.zip › cvz164-suppl_data/Supp Figure 2.tif]

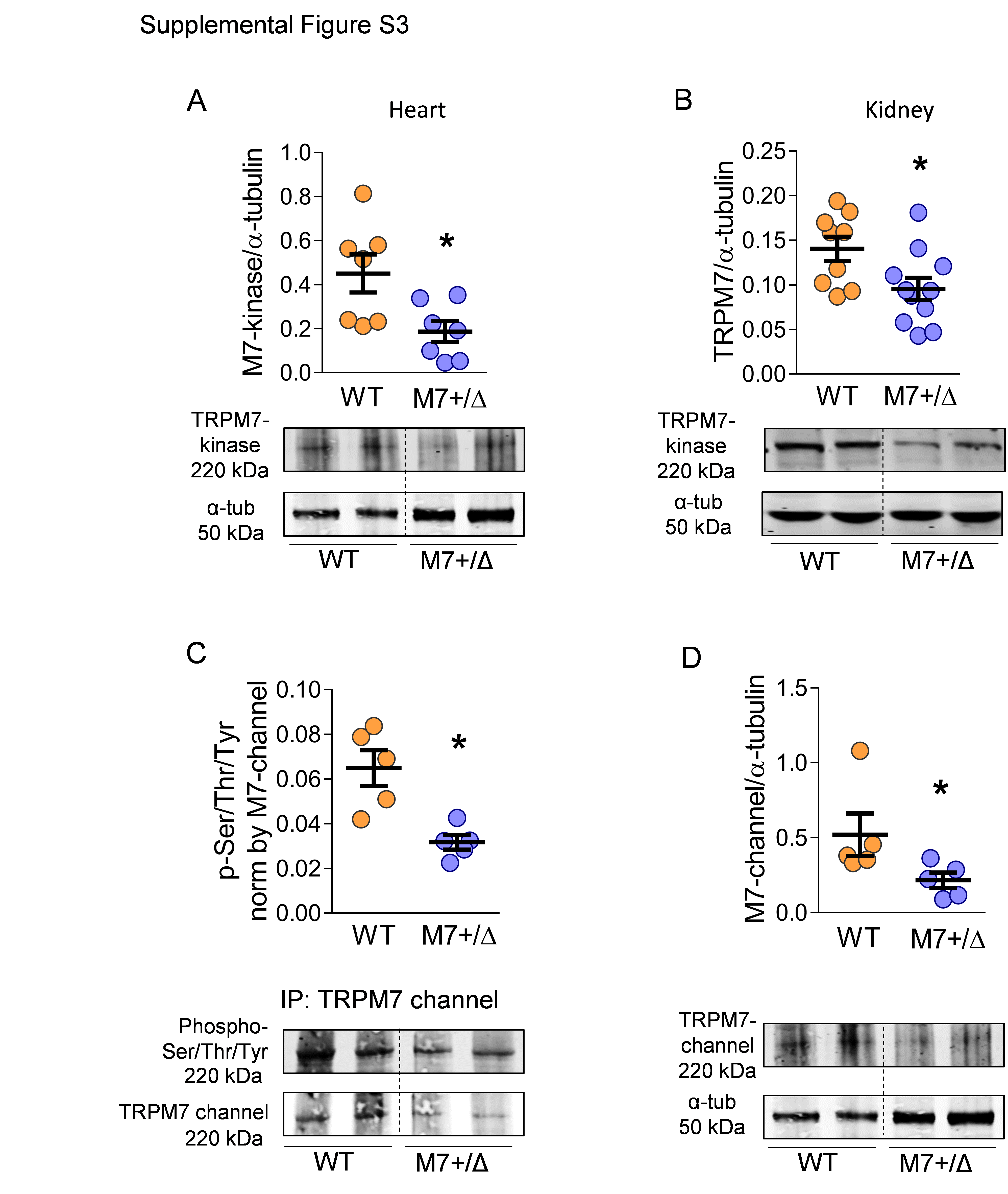

Supplement: cvz164_Supplementary_Data [file cvz164_supplementary_data.zip › cvz164-suppl_data/Supp Figure 3.tif]

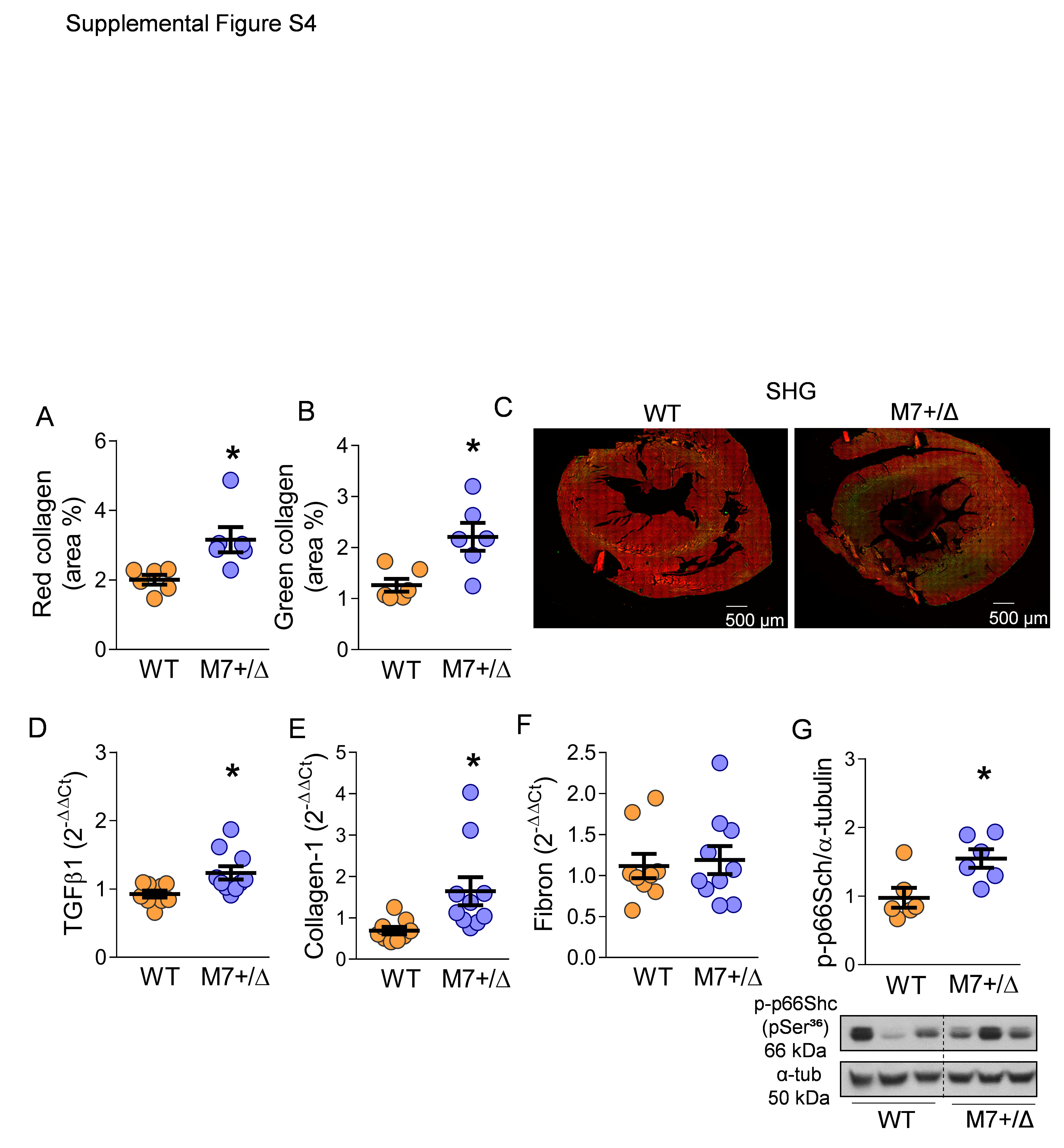

Supplement: cvz164_Supplementary_Data [file cvz164_supplementary_data.zip › cvz164-suppl_data/Supp Figure 4.tif]

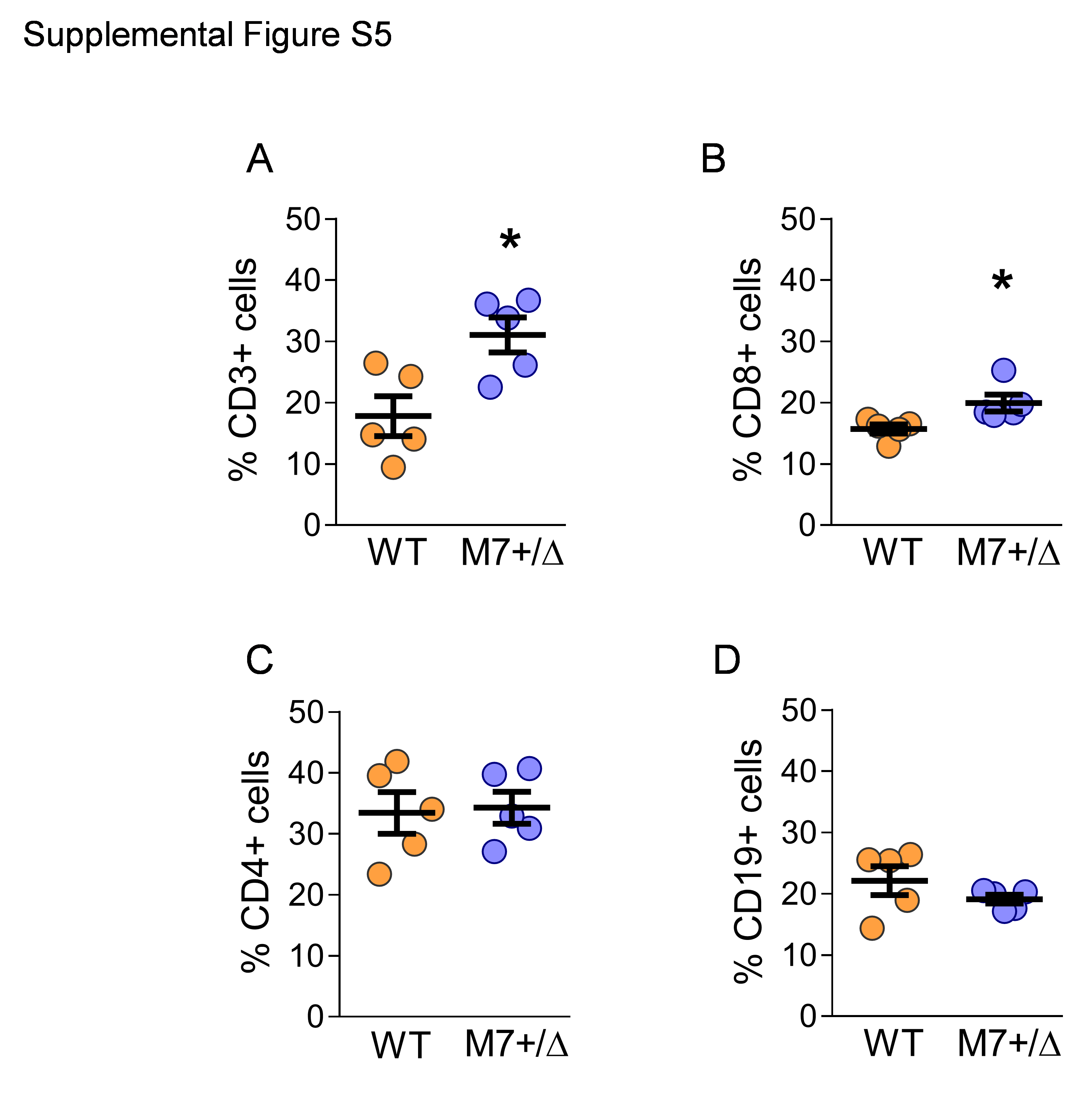

Supplement: cvz164_Supplementary_Data [file cvz164_supplementary_data.zip › cvz164-suppl_data/Supp Figure 5.tif]

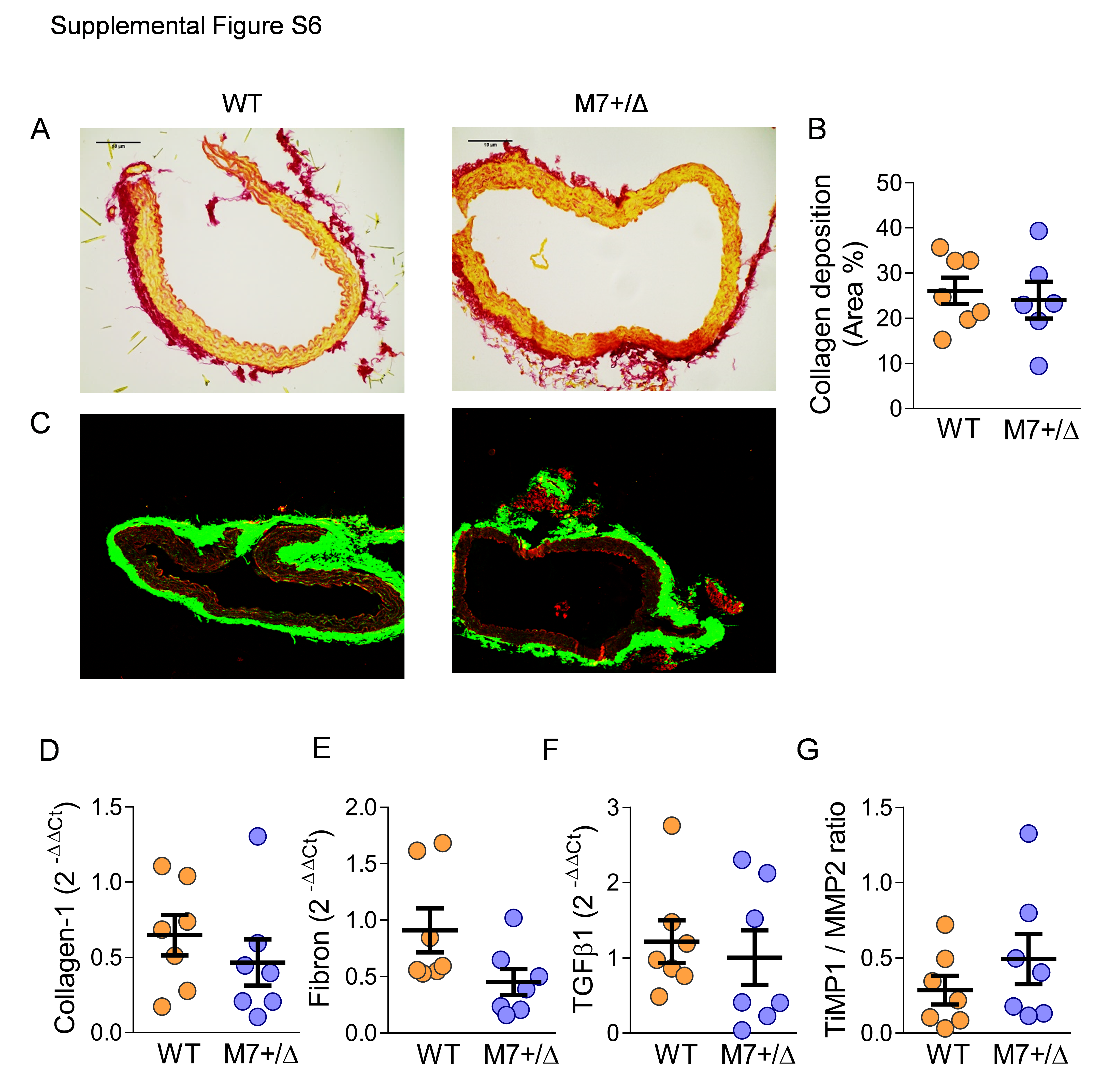

Supplement: cvz164_Supplementary_Data [file cvz164_supplementary_data.zip › cvz164-suppl_data/Supp Figure 6.tif]

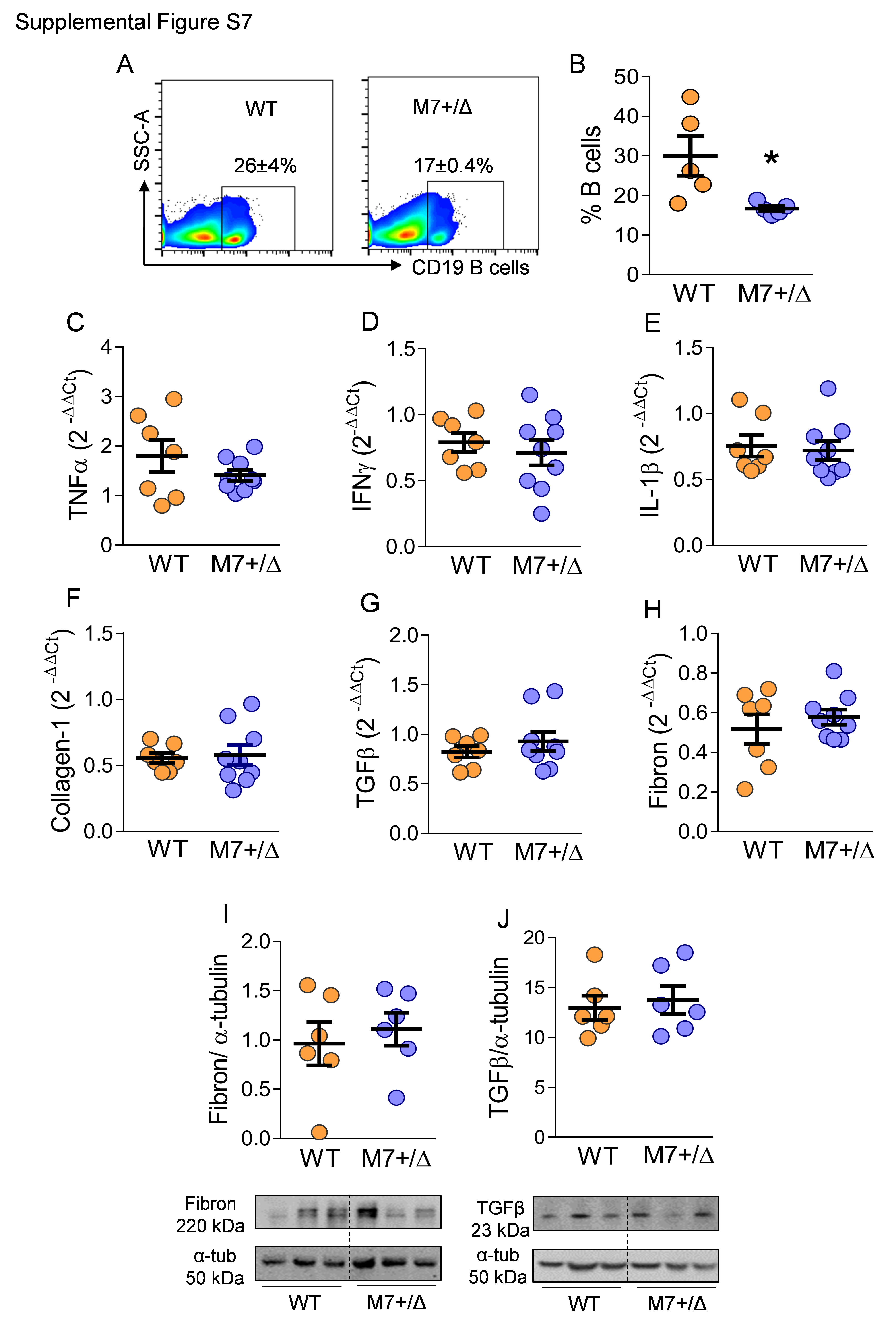

Supplement: cvz164_Supplementary_Data [file cvz164_supplementary_data.zip › cvz164-suppl_data/Supp Figure 7.tif]

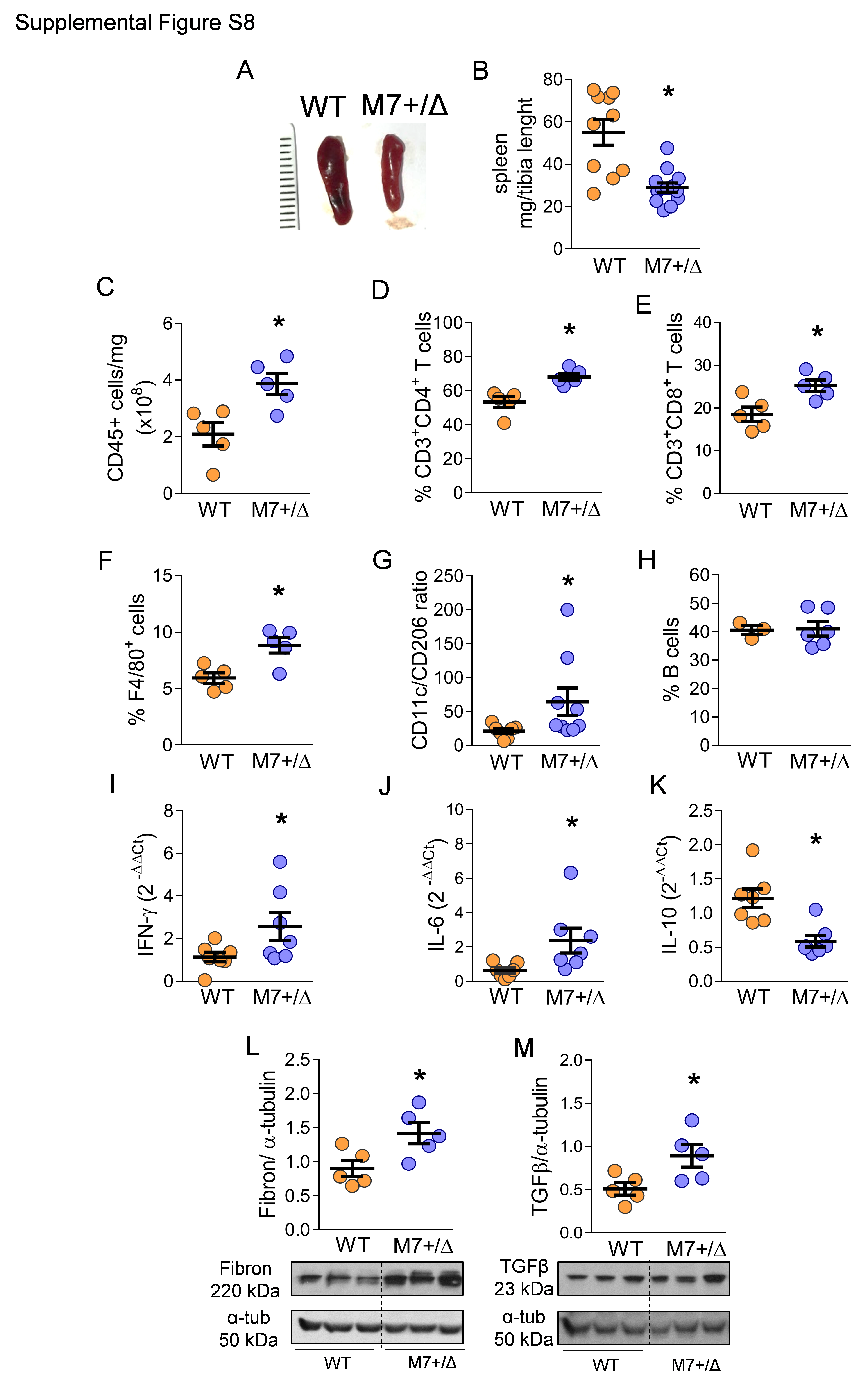

Supplement: cvz164_Supplementary_Data [file cvz164_supplementary_data.zip › cvz164-suppl_data/Supp Figure 8.tif]

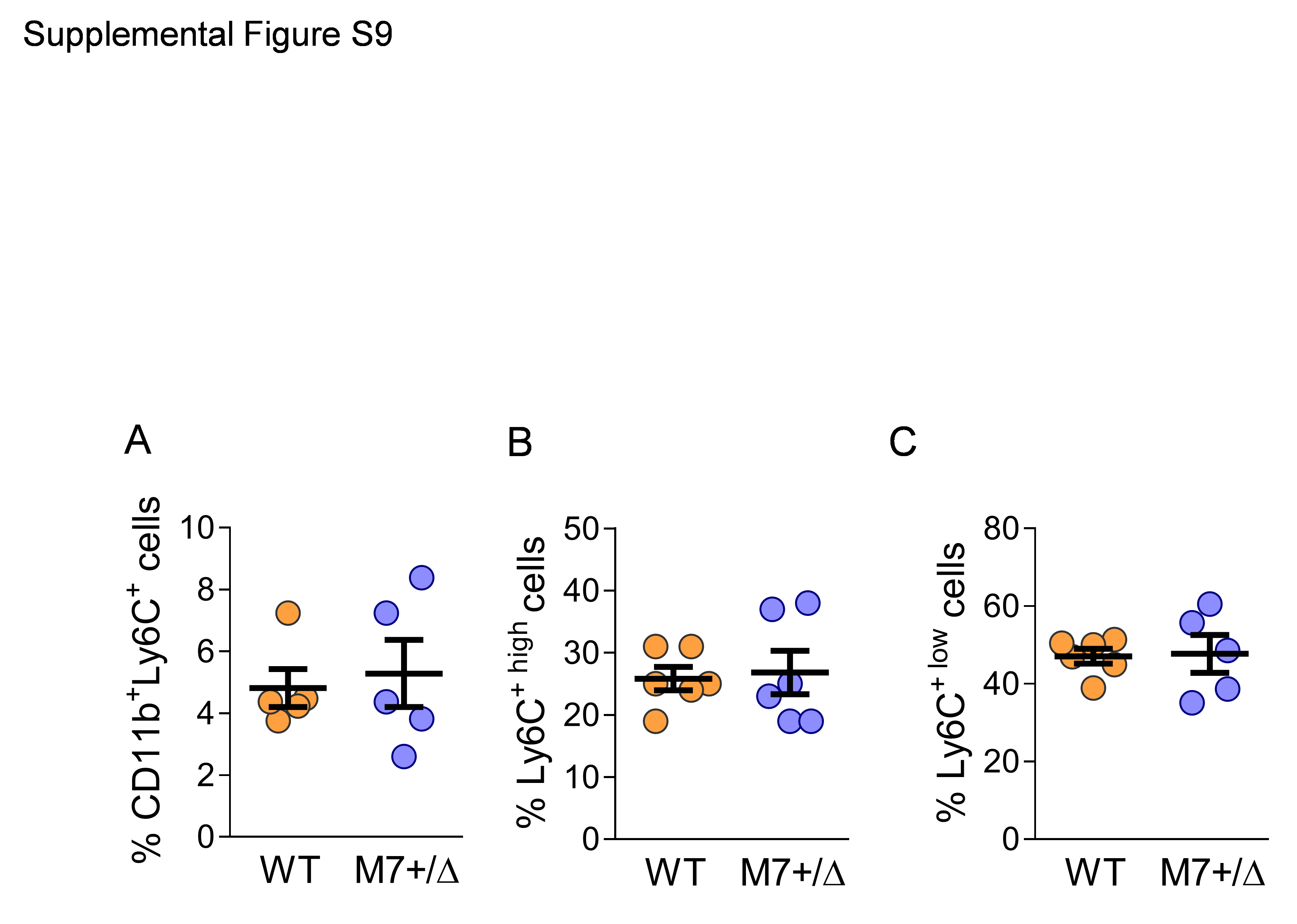

Supplement: cvz164_Supplementary_Data [file cvz164_supplementary_data.zip › cvz164-suppl_data/Supp Figure 9.tif]
